# Stochastic variational variable selection for high-dimensional microbiome data

**DOI:** 10.1186/s40168-022-01439-0

**Published:** 2022-12-24

**Authors:** Tung Dang, Kie Kumaishi, Erika Usui, Shungo Kobori, Takumi Sato, Yusuke Toda, Yuji Yamasaki, Hisashi Tsujimoto, Yasunori Ichihashi, Hiroyoshi Iwata

**Affiliations:** 1 Graduate School of Agricultural and Life Sciences, The University of Tokyo, Tokyo, Japan; 2 RIKEN BioResource Research Center, Tsukuba, Ibaraki, Japan; 3 Arid Land Research Center, Tottori University, Tottori, Japan

**Keywords:** Variational inference, Stochastic optimization, Bayesian infinite mixture model, Variable selection, Drought irrigation, Environmental and human microbiome

## Abstract

**Background:**

The rapid and accurate identification of a minimal-size core set of representative microbial species plays an important role in the clustering of microbial community data and interpretation of clustering results. However, the huge dimensionality of microbial metagenomics datasets is a major challenge for the existing methods such as Dirichlet multinomial mixture (DMM) models. In the approach of the existing methods, the computational burden of identifying a small number of representative species from a large number of observed species remains a challenge.

**Results:**

We propose a novel approach to improve the performance of the widely used DMM approach by combining three ideas: (i) we propose an indicator variable to identify representative operational taxonomic units that substantially contribute to the differentiation among clusters; (ii) to address the computational burden of high-dimensional microbiome data, we propose a stochastic variational inference, which approximates the posterior distribution using a controllable distribution called variational distribution, and stochastic optimization algorithms for fast computation; and (iii) we extend the finite DMM model to an infinite case by considering Dirichlet process mixtures and estimating the number of clusters as a variational parameter. Using the proposed method, stochastic variational variable selection (SVVS), we analyzed the root microbiome data collected in our soybean field experiment, the human gut microbiome data from three published datasets of large-scale case-control studies and the healthy human microbiome data from the Human Microbiome Project.

**Conclusions:**

SVVS demonstrates a better performance and significantly faster computation than those of the existing methods in all cases of testing datasets. In particular, SVVS is the only method that can analyze massive high-dimensional microbial data with more than 50,000 microbial species and 1000 samples. Furthermore, a core set of representative microbial species is identified using SVVS that can improve the interpretability of Bayesian mixture models for a wide range of microbiome studies.

Video Abstract

**Supplementary Information:**

The online version contains supplementary material available at 10.1186/s40168-022-01439-0.

## Background

The development of metagenomic high-throughput sequencing technologies has provided a rapid and sensitive method for the discovery of human and soil microbial communities. Accordingly, our understanding of the impact of the gut microbiota on the human body [1, 2] and the significance of bacterial ecology in the global biogeochemical nutrient cycle [3] has greatly expanded. There are two major types of microbial metagenomic data: 16S ribosomal RNA genes and shotgun metagenomics. In this study, we focus on the analysis of the 16S ribosomal RNA gene as an example, although shotgun metagenomics data can be analyzed in a relatively similar manner. One standard approach is to transform the 16S rRNA gene of the bacteria in the samples into operational taxonomic units (OTUs) using some preprocessing methods of microbiome bioinformatics platform, such as QIIME2 [4]. Using the transformed data sets, we aim to identify groups of samples based on differences in microbial composition and to elucidate the relationships between these groups.

Considering the heterogeneous pattern of sample-to-sample variability in the microbiome data, various model-based approaches have been proposed for clustering microbiome samples. The finite Dirichlet multinomial mixture (DMM) model is one of the most widely used methods [5]. The main ideas behind the DMM model are as follows. First, a multinomial sampling scheme is adopted for the taxonomic count data, and then a mixture of Dirichlet components is considered as the natural prior for the parameters of the multinomial distribution. This approach helps avoid the disadvantages of previous methods, assuming that all samples in a cohort are generated from a single community profile, and allows each community to be considered a mixture of multiple communities, which can be described by a vector generated by one of the finite Dirichlet mixture components with different hyperparameters. Therefore, the flexibility of the DMM model with respect to model dimensionality makes it well suited for capturing many different sub-community structures. The DMM model is of great practical importance and has been used to assess the potential associations of the microbiome community in studies on human health and disease [6, 7], microbiome genome-wide association [8], and animal microbiomes [9].

The first step in an analysis with a conventional DMM model is to determine the number of microbiome clusters, that is, the metacommunities biologically required to explain the observations. A fully Bayesian model selection through Laplace approximation [5] has been proposed to consider all possible values for the number of metacommunities up to a certain maximum value. However, the optimization of this number via this approach is computationally prohibitive and may cause poor performance when the dimensionality of the microbiome datasets is high. Moreover, all taxonomic units in the DMM model are considered equally significant in the clustering analysis; however, this is not realistic in practical analysis as a large number of taxonomic units may be irrelevant and would not significantly contribute to the identification (or characterization) of the microbiome clusters.

Recently, various potential approaches have been proposed to estimate the parameters of a nonparametric Bayesian unsupervised variable selection in the field of computer science, such as a typical Markov chain Monte Carlo (MCMC) approach based on either Gibbs sampling or Metropolis-Hastings algorithm that appropriately accounts for the conditional independence relationships between latent variables and model parameters [10, 11]. The MCMC approach can simulate the unobserved variables alongside the model parameters from their full posterior distribution; however, the computational burden of MCMC solutions is prohibitive for inference given the large dimensionality of microbial metagenomics datasets, and it can be very difficult to diagnose their convergence.

In this study, we propose a novel approach that overcomes the challenges described above and achieves feasible computational ability for a personal computer. The main contributions of this study are threefold. First, we propose an indicator variable that enabled the estimation of the significant contributions of taxonomic units to detect a minimum core set of taxonomic units that characterize clusters and maximize the identification ability of the clusters. Second, to overcome the current computational difficulties related to deterministic learning and MCMC approaches, we propose a stochastic variational inference (SVI) method [12–14] , which is originally used in statistical physics to approximate intractable integrals and has been successfully used in a wide variety of applications for analyzing large datasets related to population genetics [15, 16] and phylogenetics [17–19]. Moreover, we propose mathematical expansions, such as the Taylor expansion, for the special expectations that cannot be obtained directly from the analytically tractable solutions. This approach avoids expensive computations of the numerical approximations in MCMC approaches [20, 21]. Finally, we extend the finite DMM model by proposing a Bayesian nonparametric approach based on a countable infinite mixture model coupled with variable selection. In our approach, the number of clusters (metacommunities) is not fixed a priori and is itself a free parameter of inference under the truncated stick-breaking representation of the Dirichlet process prior on the mixture metacommunities [22–24]. This solution can overcome the difficulty of choosing an appropriate number of clusters based on the data.

Finally, to test the performance of the proposed approach, we use two types of 16S rRNA gene amplicon sequencing microbiome data. The first type includes several datasets in which two or three groups are known and the samples are clearly labeled. Thus, we can measure the similarity between the truth clusters and inferred clusters to compare the accuracies of the different approaches. The second type includes a dataset in which the number of groups is unknown. Various studies of the healthy human microbiome have shown that the identification of clusters (referred to as enterotypes) is difficult if the number of groups of samples is unknown [25]. Dataset A (of the first type) includes 196 and 197 rhizosphere samples from our field experiments of soybean genetic resources, which contains 888 taxonomic units from a drought irrigation and control conditions, respectively. We also apply our proposed approach to three published case-control 16S rRNA gene amplicon sequencing datasets of the human gut microbiome [26–28]. Specifically, dataset B (of the first type) includes 3347 taxonomic units for *Clostridium difficile* infection (CDI) from 338 individuals, including 89 individuals infected with CDI (cases), 89 individuals with diarrhea who test negative for CDI (diarrhea controls), and 155 non-diarrheal controls [26]. The two larger datasets of the first type are inflammatory bowel disease (IBD) data (denoted dataset C) and obesity (OB) data (denoted dataset D). These datasets provide numerous taxonomic units (approximately 10,000 and 50,000, respectively) to challenge the computational capability of stochastic variational variable selection (SVVS) [27, 28]. Dataset E (of the second type) includes the stool samples from the Human Microbiome Project (HMP), specifically the *HMP16SData* package, which has 319 samples and 11,747 taxonomic units [29], to identify the number of distinct clusters (or enterotypes).

## Materials and methods

### The finite Dirichlet multinomial mixture model

First, we briefly review the finite DMM model that describes the heterogeneity of cross-sample variability among microbiome species [5]. This model allows a dataset to be generated by a mixture of K metacommunities instead of a single metacommunity. The key concepts behind the DMM model are as follows:

Given a microbiome dataset consisting of *N* community samples and *S* taxonomic units (or species), the observed count of the $i^{th}$ community for $j^{th}$ taxonomic unit is denoted as $X_{ij}$ (*i* = 1, ..., *N*; *j* = 1, ..., *S*). The total number of counts (i.e., sequence reads) from the $i^{th}$ community sample is $J_{i} = \sum \nolimits _{j=1}^S X_{ij}$. The DMM model [5] considers a vector $\overrightarrow{X_{i}} = (X_{i1}, \ldots , X_{iS})$, drawn from a multinomial distribution with community vector $\overrightarrow{p_{i}} = (p_{i1}, \ldots , p_{iS})$ as follows:

1 $$\begin{aligned} p \left( \overrightarrow{X_{i}} | J_{i}, \overrightarrow{p_{i}} \right) \sim \mathrm {Multi} \left( J_{i}, \overrightarrow{p_{i}} \right) \end{aligned}$$

where $p_{ij}$ is the probability that a single read in the $i^{th}$ community belongs to the $j^{th}$ taxonomic unit. The DMM model defines a mixture of K Dirichlets for the multinomial parameter probability vectors $\overrightarrow{p_{i}}$. $\overrightarrow{\alpha _{k}} = (\alpha _{k1}, \ldots , \alpha _{kS})$ are the parameters of the Dirichlet distribution representing the $k^{th}$ metacommunity (or cluster), and $\boldsymbol{\pi } = (\pi _{1}, \ldots , \pi _{K})$ represents the mixing coefficients with $\sum \nolimits _{k=1}^{K} \pi _k = 1$, $\pi _k \ge 0$ for $k \in (1, \ldots , K)$. The finite DMM model examines a case where the number of metacommunities, K, is fixed. Each sample is assumed to be drawn from each unique community vector $\overrightarrow{p_{i}}$, which is derived from one of the K metacommunities. The DMM model introduces the allocation variable $\overrightarrow{Z_{i}} = (Z_{i1}, Z_{i2}, \ldots , Z_{ik})$, where $Z_{ik} \in \{0,1\}$ and $\sum \nolimits _{k=1}^K Z_{ik} = 1$. If $\overrightarrow{X_{i}}$ belongs to the $k^{th}$ metacommunity (i.e., the $k^{th}$ cluster), then the value of $Z_{ik}$ is one; otherwise, it is zero. The distribution of **Z** follows the categorical distribution $p \left( \overrightarrow{Z_{i}} | \boldsymbol{\pi } \right) = \prod _{k=1}^K \pi _k^{Z_{ik}}$. Therefore, Eq. (1) can be rewritten by marginalizing the multinomial parameters as follows [5]:

2 $$\begin{aligned} p \left( \boldsymbol{X} | \boldsymbol{Z}, \boldsymbol{\alpha } \right) = \prod\limits_{i=1}^N \prod\limits_{k=1}^K \left( \frac{B \left( \overrightarrow{\alpha _k} + \overrightarrow{X_{i}} \right) }{B \left( \overrightarrow{\alpha _k} \right) } J_{i}! \prod\limits_{j=1}^S \frac{1}{X_{ij}!} \right) ^{Z_{ik}} \end{aligned}$$

where the function B is the multinomial beta function $B \left( \overrightarrow{\alpha _k} \right) = \frac{\prod _{j=1}^S \Gamma (\alpha _{kj})}{\Gamma \left( \sum \nolimits _{j=1}^S \alpha _{kj} \right) }$ and $B \left( \overrightarrow{\alpha _k} + \overrightarrow{X_{i}} \right) = \frac{\prod _{j=1}^S \Gamma (\alpha _{kj} + X_{ij})}{\Gamma \left( \sum \nolimits _{j=1}^S \left( \alpha _{kj} + X_{ij} \right) \right) }$

### The infinite Dirichlet multinomial mixture model with variable selection

The goal is to consider the number of metacommunities (*K*) as a random variable. To achieve this, it is assumed that the prior distribution of the mixing coefficients $\boldsymbol{\pi }$ follows a Dirichlet process prior [22]. The stick-breaking representation [23, 24], which is a straightforward constructive definition of the Dirichlet process, is adopted to construct the infinite DMM model proposed in this study. This is defined as follows:

$$\begin{aligned}&p \left( \gamma _{k} \right) \sim \mathrm {Beta} \left( 1, \nu \right) \\&\pi _{k} = \gamma _{k} \prod\limits_{k'=1}^{k-1}(1 - \gamma _{k'}) \end{aligned}$$

where $\pi _{k}$ is the mixing proportion of an infinite number of successively broken sticks, and independent random variables $\gamma _{k}$ with $\left( k \in \left[ 1, \ldots , K \right] \right)$ represent proportions that are sequentially broken from the remaining length, $\prod _{k'=1}^{k-1}(1 - \gamma _{k'})$, of a unit-length stick, and $\nu$ represents the total mass parameter of the Dirichlet process. It is assumed that each community sample $\overrightarrow{X_{i}}$ is generated from the DMM model with a countably infinite of number of clusters (or metacommunities). Therefore, the Eq. (2) can be rewritten as

3 $$\begin{aligned} p \left( \boldsymbol{X} | \boldsymbol{Z}, \boldsymbol{\alpha } \right) = \prod\limits_{i=1}^N \prod\limits_{k=1}^{\infty } \left( \frac{B \left( \overrightarrow{\alpha _k} + \overrightarrow{X_{i}} \right) }{B \left( \overrightarrow{\alpha _k} \right) } J_{i}! \prod\limits_{j=1}^S \frac{1}{X_{ij}!} \right) ^{Z_{ik}} \end{aligned}$$

All taxonomic units in the DMM model are assumed to be equally important for clustering microbial community data. However, this is not realistic in microbiome studies, because numerous microbiome species (which can be reflected in taxonomic units) and functions might be irrelevant and significantly influence the performance of clustering algorithms [30]. To overcome this problem, we propose that the count of a given taxonomic unit, $X_{ij}$, be generated from a mixture of two Dirichlet-multinomial distributions; the first one is assumed to generate a core set of the most significant microbial taxonomic units and is different for each metacommunity (i.e., each cluster), and the second one is assumed to generate the unimportant taxonomic units and was common to all metacommunities (i.e., all clusters). Thus, we can write the likelihood of the observed microbiome dataset $\boldsymbol{X}$ following the infinite DMM model with microbiome taxonomic unit selection as follows:

4 $$\begin{aligned} p \left( \boldsymbol{X} | \boldsymbol{Z}, \boldsymbol{\phi }, \boldsymbol{\alpha }, \boldsymbol{\beta } \right) = \prod\limits_{i=1}^N \prod\limits_{k=1}^{\infty } \left[ \begin{array}{c} \left( \frac{B \left( \overrightarrow{\alpha _k} + \overrightarrow{X_{i}} \right) }{B \left( \overrightarrow{\alpha _k} \right) } J_{i}! \prod\limits_{j=1}^S \frac{1}{X_{ij}!} \right) ^{\phi _{ij}}\\ \left( \frac{B \left( \boldsymbol{\beta } + \overrightarrow{X_{i}} \right) }{B \left( \boldsymbol{\beta } \right) } J_{i}! \prod\limits_{j=1}^S \frac{1}{X_{ij}!} \right) ^{1 - \phi _{ij}} \end{array}\right] ^{Z_{ik}} \end{aligned}$$

where $\phi _{ij}$ is an indicator variable, such that $\phi _{ij} = 1$ indicates that the $j^{th}$ taxonomic unit of the $i^{th}$ community is important for clustering and follows a Dirichlet multinomial distribution with $\boldsymbol{\alpha }$, and $\phi _{ij} = 0$ denotes that the $j^{th}$ taxonomic unit of $i^{th}$ the community is unimportant for clustering and follows a Dirichlet multinomial distribution with $\boldsymbol{\beta }$. $\phi _{ij}$ characterizes the importance of each taxonomic unit in a sample. Although some samples are assigned to a cluster, each sample has a different group of important taxonomic units that are selected in the clustering process. $B \left( \overrightarrow{\alpha _k} \right)$ and $B \left( \overrightarrow{\alpha _k} + \overrightarrow{X_{i}} \right)$ are the multinomial beta functions for a core set of taxonomic units that significantly represent the cluster. For unimportant species, the multinomial beta functions are $B \left( \boldsymbol{\beta } \right) = \frac{\prod _{j=1}^S \Gamma (\beta _{j})}{\Gamma \left( \sum \nolimits _{j=1}^S \beta _{j} \right) }$ and $B \left( \boldsymbol{\beta } + \overrightarrow{X_{i}} \right) = \frac{\prod _{j=1}^S \Gamma (\beta _{j} + X_{ij})}{\Gamma \left( \sum \nolimits _{j=1}^S \left( \beta _{j} + X_{ij} \right) \right) }$. The prior distribution of the indicator variable of microbiome selection $\boldsymbol{\phi }$ is defined as follows:

$$\begin{aligned} p \left( \boldsymbol{\phi } |\boldsymbol{\epsilon } \right) = \prod\limits_{i=1}^N \prod\limits_{j=1}^S \epsilon _{j_{1}}^{\phi _{ij}} \epsilon _{j_{2}}^{1 - \phi _{ij}} \end{aligned}$$

where each $\phi _{ij}$ follows a Bernoulli distribution such that $p \left( \phi _{ij} = 1\right) = \epsilon _{j_{1}}$ and $p \left( \phi _{ij} = 0\right) = \epsilon _{j_{2}}$ with $\epsilon _{j_{1}} + \epsilon _{j_{2}} = 1$ [11]. Furthermore, we use the Beta distributions over $\boldsymbol{\epsilon }$ [31].

$$\begin{aligned} p \left( \boldsymbol{\epsilon } |\boldsymbol{\xi } \right) = \prod\limits_{j=1}^S \frac{\Gamma (\xi _{1} + \xi _{2})}{\Gamma (\xi _{1}) \Gamma (\xi _{2})} \epsilon _{j_{1}}^{\xi _{1}-1} \epsilon _{j_{2}}^{\xi _{2}-1} \end{aligned}$$

where the hyperparameters $(\xi _{1}, \xi _{2})>0$ are subject to the constraint in order to ensure that the distribution can be normalized. The prior distributions of $\boldsymbol{\alpha }$ and $\boldsymbol{\beta }$ follow the Dirichlet distributions with hyperparameters $\boldsymbol{\zeta }$ and $\boldsymbol{\eta }$.

$$\begin{aligned} p \left( \boldsymbol{\alpha } |\boldsymbol{\zeta } \right) = \prod\limits_{k=1}^{\infty } \frac{\Gamma \left( \sum \nolimits _{j=1}^S \zeta _{kj} \right) }{ \prod _{j=1}^S \Gamma (\zeta _{kj})} \prod\limits_{j=1}^S \alpha _{kj}^{\zeta _{kj}-1} \end{aligned}$$

$$\begin{aligned} p \left( \boldsymbol{\beta } |\boldsymbol{\eta } \right) = \frac{\Gamma \left( \sum \nolimits _{j=1}^S \eta _{j} \right) }{ \prod _{j=1}^S \Gamma (\eta _{j})} \prod\limits_{j=1}^S \beta _{j}^{\eta _{j}-1} \end{aligned}$$

In our computational experiments, we attempted to use both Gamma distribution and Dirichlet distribution for the prior distributions for $\boldsymbol{\alpha }$ and $\boldsymbol{\beta }$. However, scale parameter of Gamma distribution was not able to obtain good updated values. Parameters of Dirichlet distributions obtained the better updated values for each iteration; therefore, we opted to choose Dirichlet distributions.

### Stochastic variational variable selection approach

In this section, we propose an SVI method [12–14] for performing the infinite DMM model with feature selection. The basic idea of variational learning in the Bayesian approach is to approximate the posterior distribution using a computationally tractable function called the variational distribution. The variational parameter, which specifies the variational distribution, is estimated by minimizing the Kullback-Leibler (KL) divergence of the posterior distribution to the variational distribution. As a result, the posterior distribution is estimated by numerical optimization without invoking the simulation approaches, such as MCMC algorithms.

Given the observed count dataset $\boldsymbol{X}$, the infinite DMM model has a set of parameters $(\Xi )$, which consists of the stick-breaking proportions $(\boldsymbol{\gamma })$, the allocation variable $(\boldsymbol{Z})$ of the prior Dirichlet, the indicator variable of the taxonomic unit selection $(\boldsymbol{\phi })$, and the Dirichlet parameters $(\boldsymbol{\alpha , \beta })$. At the initial step of the variational approach, we propose an element of a tractable family of probability distributions $q \left( \Xi | \Theta \right)$ called the variational distribution, which approximates the true intractable posterior distribution $p \left( \Xi | \boldsymbol{X} \right)$. This distribution is parameterized by free parameters, called variational parameters $\Theta$.

Subsequently, variational inference estimates these parameters to find a distribution close to the true intractable posterior distribution of interest. The distance between the distributions $p \left( \Xi | \boldsymbol{X} \right)$ and $q \left( \Xi | \Theta \right)$ is evaluated using KL divergence, defined as follows:

5 $$\begin{aligned}&\mathrm {KL} \left[ q \left( \Xi | \Theta \right) | p \left( \Xi | \boldsymbol{X} \right) \right] \nonumber \\&\quad = \mathrm {E_{q}} \left[ \mathrm {log} \left( q \left( \Xi | \Theta \right) \right) \right] - \mathrm {E_{q}} \left[ \mathrm {log} \left( p \left( \Xi | \boldsymbol{X} \right) \right) \right] \nonumber \\&\quad = \mathrm {E_{q}} \left[ \mathrm {log} \left( q \left( \Xi | \Theta \right) \right) \right] - \mathrm {E_{q}} \left[ \mathrm {log} \left( p \left( \Xi , \boldsymbol{X} \right) \right) \right] + \mathrm {log} \left( p \left( \boldsymbol{X} \right) \right) \end{aligned}$$

The log marginal probability $\mathrm {log} \left( p \left( \boldsymbol{X} \right) \right)$ in Eq. (5), which causes computational difficulty in the Bayesian approach, can be treated as a constant term in the numerical optimization for estimating the variational parameters as follows:

$$\begin{aligned} \Theta ^* = \mathrm {argmin KL} \left[ q \left( \Xi | \Theta \right) | p \left( \Xi | \boldsymbol{X} \right) \right] \end{aligned}$$

In addition, the term $\mathrm {log} \left( p \left( \boldsymbol{X} \right) \right)$, which is known as the evidence of $\boldsymbol{X}$, can be decomposed as $\mathrm {log} \left( p \left( \boldsymbol{X} \right) \right) = \mathcal {L} \left[ q \left( \Xi | \Theta \right) \right] + \mathrm {KL} \left[ q \left( \Xi | \Theta \right) | p \left( \Xi | \boldsymbol{X} \right) \right]$. The variational inference maximizes the computationally feasible target function defined as:

6 $$\begin{aligned} \mathcal {L} \left[ q \left( \Xi | \Theta \right) \right] = \mathrm {E_{q}} \left[ \mathrm {log} \left( p \left( \Xi , \boldsymbol{X} \right) \right) \right] - \mathrm {E_{q}} \left[ \mathrm {log} \left( q \left( \Xi | \Theta \right) \right) \right] \end{aligned}$$

where Eq. (6) is the Evidence Lower Bound (ELBO) [12]. $\mathcal {L} \left[ q \left( \Xi | \Theta \right) \right]$ can be considered a lower bound for $\mathrm {log} \left( p \left( \boldsymbol{X} \right) \right)$. The maximization of ELBO equals the minimization of KL divergence, that is, when the variational distribution $q \left( \Xi | \Theta \right)$ approximates the true posterior distribution $p \left( \Xi | \boldsymbol{X} \right)$. However, direct application of the variational approach is unfeasible. Therefore, a mean-field approach is adopted in order to factorize the posterior distribution into disjoint tractable distributions. According to the factorization assumption of mean-field variational approximations [13, 14], each variable in the variational distribution $q \left( \Xi | \Theta \right)$ is independent. Furthermore, we use truncated stick-breaking representations to approximate the posterior Dirichlet process. The truncation level $\mathrm {K}$ is not a part of the prior model specification. The variational approach can optimize the value of $\mathrm {K}$ because it becomes a variational parameter [13, 32, 33]. The family of variational distributions in the infinite DMM model with the selection of representative taxonomic units can be expressed as follows:

7 $$\begin{aligned}&q \left( \boldsymbol{Z, \phi , \gamma , \epsilon , \alpha , \beta } | \Theta \right) \nonumber \\&=\prod\limits_{i=1}^{N} \prod\limits_{k=1}^{{K}} q \left( Z_{ik} \right) \times \prod\limits_{k=1}^{{K}} q \left( \gamma _{k} \right) \times \prod\limits_{i=1}^{N} \prod\limits_{j=1}^{S}q \left( \phi _{ij} \right) \times q \left( \boldsymbol{\epsilon } \right) \times q \left( \boldsymbol{\alpha } \right) \times q \left( \boldsymbol{\beta } \right) \end{aligned}$$

where

8 $$\begin{aligned} q \left( \boldsymbol{Z} \right)= & {} \prod\limits_{i=1}^{N} \prod\limits_{k=1}^{{K}} r_{ik}^{Z_{ik}} \nonumber \\ q \left( \boldsymbol{\phi } \right)= & {} \prod\limits_{i=1}^{N} \prod\limits_{j=1}^{S} f_{ij}^{\phi _{ij}} \left( 1 - f_{ij} \right) ^{1-\phi _{ij}} \nonumber \\ q \left( \boldsymbol{\gamma } \right)\sim & {} \prod\limits_{k=1}^{{K}} \mathrm {Beta} \left( \gamma _{k} | \vartheta _{k}, \vartheta _{k}^{'} \right) \nonumber \\ q \left( \boldsymbol{\epsilon } \right)\sim & {} \mathrm {Dirichlet} \left( \boldsymbol{\epsilon } | \boldsymbol{\xi ^{*}} \right) \nonumber \\ q \left( \boldsymbol{\alpha } \right)\sim & {} \mathrm {Dirichlet} \left( \boldsymbol{\alpha } | \boldsymbol{\lambda ^{*}} \right) \nonumber \\ q \left( \boldsymbol{\beta } \right)\sim & {} \mathrm {Dirichlet} \left( \boldsymbol{\beta } | \boldsymbol{\iota ^{*}} \right) \end{aligned}$$

The set of free variational parameters $\Theta$ includes $\boldsymbol{r}, \boldsymbol{\vartheta }, \boldsymbol{\vartheta {'}}, \boldsymbol{f}, \boldsymbol{\xi ^{*}}, \boldsymbol{\lambda ^{*}}, \boldsymbol{\iota ^{*}}$. We use the variational distributions from exponential families to guarantee tractable computations of expectations.

The key idea of SVI inference is to divide the variational variables into two subgroups: the local variables $\left[ \Xi _l \in \left( \boldsymbol{Z, \phi } \right) \right]$, which are per-datapoint latent variables, and the global variables $\left[ \Xi _g \in \left( \boldsymbol{\gamma , \epsilon , \alpha , \beta } \right) \right]$, which potentially control all the data. The $i^{th}$ local variable $Z_{ik}$ of the mixture component, which represents the allocation of sample *i*, is governed by the local variational parameter $r_{ik}$. In addition, the local variational parameter $f_{ij}$ is proposed to capture the $i^{th}$ local variable $\phi _{ij}$, which represents the selection situation of the $j^{th}$ taxonomic unit in the $i^{th}$ community. The coordinate ascent algorithm is used to overcome the optimization problems of these variational variables [13, 14]. The main idea of this approach is to optimize each factor of the mean-field variational distribution while fixing the others. For example, we obtain the optimal solution of local variable $Z_{ik}$ by applying variational distributions in Eqs. (7) and (8) to the ELBO in Eq. (6). We omit terms that do not depend on the variational parameter of $Z_{ik}$. The logarithm of the optimal value of $q \left( Z_{ik} \right)$ is proportional to the expected logarithm of the joint distribution as follows:

9 $$\begin{aligned}&\mathrm {log} q^{*} \left( Z_{ik} \right) \nonumber \\&\propto \sum\limits_{j=1}^S \mathrm {E}_{q} \left[ \phi _{ij} \right] \left( \mathrm {E}_{q} \left[ \mathrm {log} \left( \frac{ \Gamma \left( \sum_{j=1}^S \alpha _{kj} \right) }{\Gamma \left( \sum _{j=1}^S X_{ij} + \sum _{j=1}^S \alpha _{kj} \right) } \right) \right] \right) \nonumber \\&+ \sum\limits_{j=1}^S \mathrm {E}_{q} \left[ \phi _{ij} \right] \left( \mathrm {E}_{q} \left[ \mathrm {log} \left( \frac{\Gamma \left( X_{ij} + \alpha _{kj}\right) }{\Gamma (\alpha _{kj})} \right) \right] \right) \nonumber \\&+ \sum\limits_{j=1}^S \mathrm {E}_{q} \left[ \phi _{ij} \right] \left( \mathrm {log} (J_{i}!) + \mathrm {log} \left( \frac{1}{X_{ij}!} \right) \right) \nonumber \\&+ \mathrm {E}_{q} \left[ \mathrm {log} (\gamma _{k}) \right] + \sum\limits_{k'=1}^{k-1} \mathrm {E}_{q} \left[ \mathrm {log} (1-\gamma _{k'}) \right] \end{aligned}$$

As $\gamma _k$ follows a beta distribution, we can obtain the analytically tractable solutions for $\mathrm {E}_{q} \left[ \mathrm {log} (\gamma _{k}) \right]$ and $\mathrm {E}_{q} \left[ \mathrm {log} (1-\gamma _{k'}) \right]$. However, the first and second terms of Eq. (9) do not have the same form as the logarithm of the Dirichlet prior distribution. Thus, analytically tractable solutions cannot be obtained directly. The intractable computation of expectations can be resolved using the Metropolis-Hastings algorithm and numerical integration. Nevertheless, the simulation approaches significantly increase the computational burden in the huge dimensionality of microbial metagenomics datasets [5]. Therefore, we adopt the Taylor expansion to obtain the nearly optimal analytically tractable solutions for the first and second terms of Eq. (9), such that the computational burdens are avoided [20, 21, 34]. A nearly optimal analytically tractable value of $q \left( \phi _{ij} \right)$ can be obtained using the proposed approach. The mathematical details of the Taylor expansion and variational objective functions are provided in the Supplementary Material.

The global variational parameters $\left[ \Theta _{g} \in \left( \vartheta _{k}, \vartheta _{k}^{'}, \xi ^{*}, \lambda _{kj}^{*}, \iota _{j}^{*} \right) \right]$ are proposed to govern the global variable $\Xi _g$. The SVI approach uses the stochastic gradient ascent to estimate the global variational parameters [14]. This is mainly because as the sizes of microbiome datasets increase, each iteration of coordinate ascent algorithm becomes more computationally expensive. The computational structure of the algorithm therefore requires iterating over the entire dataset for each iteration. The SVI, however, is based on the stochastic approximation approach that iteratively generates subsampled datasets that are used to update the values of the local and global variational parameters. The main advantage of these computational strategies is that they ensure that algorithms will avoid shallow local optima for complex objective functions. Furthermore, the natural gradients are an important part of the SVI approach that increase the scale of variational inference and allow for the analysis of vast amounts of data [35–37]. Natural gradients adjust the direction of the conventional gradients to account for the geometric structure of probability parameters that use the Riemannian metric and the Fisher information matrix. Therefore, the natural gradients are not only cheaper computations but also have faster convergence than conventional gradients.

Principally, we seek to construct a noisy but unbiased and cheap-to-compute natural gradient to reach the optimum of the objective function of the infinite DMM model. First, we generate a uniform a dataset $\left[ \overrightarrow{X_{n}}^{(N)}, \overrightarrow{Z_{n}}^{(N)}, \overrightarrow{\phi _{n}}^{(N)}\right]$ that is formed by *N* replicated from the microbiome community sample $\overrightarrow{X_{i}}$, allocation variable $\overrightarrow{Z_{i}}$, and indicator variable $\overrightarrow{\phi _{i}}$ at each iteration. Next, noisy estimates of the natural gradient are computed with respect to each global variational parameter $\Theta _g$ given N replicates of the sampled data point. Using these gradients, the values of $\Theta _g$ are updated at iteration *m* given the local variational parameters $\left[ \Theta _{l} \in \left( r_{ik}, f_{ij} \right) \right]$ as follows:

$$\begin{aligned} \widehat{\nabla _{\Theta _{g}}} \mathcal {L}= & {} \mathrm {prior} + N \left( \mathrm {E}_{ \Theta _{l}} \left[ t \left( \overrightarrow{X_{n}}, \overrightarrow{Z_{n}}, \overrightarrow{\phi _{n}} \right) , 1\right] \right) - \Theta _{g} \nonumber \\ \Theta _{g}^{(m)}= & {} \Theta _{g}^{(m-1)} + \rho _m \widehat{\nabla _{\Theta _{g}}} \mathcal {L} \end{aligned}$$

where t(.) denotes the sufficient statistics in the exponential family and $\rho _m$ denotes the step size at iteration *m*. Owing to the subsampling strategies, the SVI significantly accelerates the computational processes by avoiding expensive sums in the ELBO when the dimensionality of the microbial metagenomics is large. The mathematical explanations of the SVI are described in the Supplementary Material.

### Criteria to evaluate the performance of the approaches

We use the Adjusted Rand Index (ARI) [38] in order to measure the similarity between the truth (or known) clusters and clusters inferred by various algorithms. Given a dataset of $\boldsymbol{X}$ with n total samples, $\boldsymbol{Z} = \left[ Z_1, \dots , Z_k \right]$ denotes the true cluster memberships of $\boldsymbol{X}$ into k clusters, and $\boldsymbol{Z'} = \left[ Z'_1, \dots , Z'_{k'} \right]$ denotes an inferred cluster membership of $\boldsymbol{X}$ into k’ clusters. The Rand Index (RI) is calculated as follows:

$$\begin{aligned} R \left( \boldsymbol{Z, Z'} \right) = \frac{a+b}{n \left( n-1 \right) /2} \end{aligned}$$

where *a* denotes the number of times a pair of samples is assigned to the same cluster in $\boldsymbol{Z}$ and $\boldsymbol{Z'}$, and *b* denotes the number of times a pair of samples is assigned to different clusters in $\boldsymbol{Z}$ and $\boldsymbol{Z'}$. The RI values are in the range of [0,1], where 1 represents a perfect similarity between the truth and inferred clusters. The ARI is proposed to normalize the difference between the RI and its expected value as follows:

$$\begin{aligned} \mathrm {ARI} = \frac{RI - \mathrm {E} (RI)}{\max (RI) - \mathrm {E} (RI)} \end{aligned}$$

where $\mathrm {E} (RI)$ is the expected value of the RI.

### Database description

#### Study inclusion and data acquisition

Dataset A represents the environmental microbiome data of our field experiments, which includes 196 drought irrigation samples, 197 control conditions samples and 888 microbiome species (or taxonomic units). The experimental explanations of dataset A are described in the Supplementary Material.

We also employ case-control 16S amplicon sequencing from three published human microbiome datasets spanning three different disease states: *Clostridium difficile* infection (CDI) [26], inflammatory bowel disease (IBD) [28], and obesity (OB) [27]. These datasets are available in the MicrobiomeHD database [39]. Dataset B represents the CDI dataset, which includes 183 diarrheal stool samples from the 94 individuals with CDI, 89 diarrheal control samples, 155 non-diarrheal control stool samples, and 3347 microbiome species (or taxonomic units). Dataset C represents the IBD dataset, which includes 146 IBD case samples, 16 non-IBD control samples and 10,119 microbiome species (or taxonomic units). Dataset D representes the OB dataset, which is the largest and most challenging. There are 1081 fecal samples from 977 individuals and 55,964 microbiome species (or taxonomic units).

Finally, we use the variable region 3-5 (V35) of the 16S rRNA gene sequence dataset from the *HMP16SData* package in R to study the considerable variation in the composition of the healthy human microbiome [29]. Dataset E represents the data of stool community types from the *HMP16SData* package, which includes 319 samples and 11,747 microbiome species (or taxonomic units). Moreover, we use other R packages to perform the graphical visualizations for the microbiome datasets, such as the unweighted UniFrac distance and non-metric multidimensional scaling (NMDS) functions in the *phyloseq* package [40].

### Open-source software

The software is implemented in Python and used standard libraries, such as NumPy and SciPy, for mathematical computations. The software inputs microbiome count data in a CSV file and outputs the inferred clusters and a core set of selected taxonomic units. The main options in the software tool are the maximum number of clusters, which pose limitations in estimating the number of clusters, and the number of taxonomic units that users want to select. SVVS uses the iterative optimization algorithms to estimate the parameters; thus, a convergence criterion is used to implement a stopping rule. The SVVS algorithm stops when the change in the ELBO computations is less than 1e−3 (Supplementary Material). We use the convergence criterion fixed across all datasets in this study. The number of iterations should be modified for datasets notably smaller or larger in scale than those considered in this study. This is a tunable option in the software. The software is available at https://github.com/tungtokyo1108/SVVS.

In all our experiments, we initialized the truncation levels of the number of clusters to 10. We set the initial values of hyperparameter $\nu$ of the stick-breaking representation to 0.1, the initial values of hyperparameters $\boldsymbol{\zeta }$ and $\boldsymbol{\eta }$ of the Dirichlet priors to 1, and those of hyperparameters $(\xi _{1}, \xi _{2})$ to 0.1 [41].

To address the selection of species based on the model, we calculate average of $\phi _{ij}$ over sample *i* after estimating the values of $\phi _{ij}$ and ranked microbiome species from the highest to lowest values. Our package exports a table containing these ranked values, and a user can then select a core set of microbial species from the higher values in this table. For example, Tables S1 and S2 show the average values of $\phi _{ij}$ over sample *i* that are arranged in descending order (largest first) in the dataset A and B.

## Results

### Runtime performance and physical memory of the computational system

An important advantage of SVVS over conventional DMM approaches is that the computational time and memory required for calculations can be greatly reduced. To evaluate the computational time and memory of the different approaches, we varied the sample size and number of taxonomic units in the sample datasets. The scalability of the methods was specifically demonstrated in cases of datasets C and D; meanwhile, datasets A and B were selected to compare their accuracies. We followed the Laplace approximation to the model evidence and default values of the DirichletMultinomial 1.34.0 package in R to determine the number of clusters K for the finite DMM model [5, 42]. Our proposed method does not require selection of the number of clusters because the number of clusters is estimated as a random variable. Our Python implementations of SVVS for the infinite DMM model were used to analyze all empirical datasets. Tables 1 and 2 compare the computational time and physical memory required for the calculation between the SVVS algorithm of the infinite DMM model and the EM algorithm of the finite DMM model. We found that SVVS was able to considerably reduce run times and physical memories for datasets A, B, and C. SVVS was the only approach that was able to analyze dataset D, which is a large dataset of more than 50,000 taxonomic units and 1,000 samples. In addition, the computational time and memory of each of the above methods were found to significantly increase with the number of taxonomic units and samples.

**Table 1:** Running time of the two approaches on the empirical datasets. Note: All algorithms were run on a personal computer (Intel$\circledR$ Xeon$\circledR$ Gold 6230 Processor 2.10 GHz $\times$ 2, 40 cores, 2 threads per core, 128 Gb RAM) under Ubuntu 20.04.1 LTS

Datasets	Finite DMM with EM algorithm	Infinite DMM with SVVS algorithm
A	17.63 min	2.68 min
B	2.75 h	13.25 min
C	3.37 d	30 min
D	Failed	5 h

**Table 2:** Physical memories of the two approaches on the empirical datasets

Datasets	Finite DMM with EM algorithm	Infinite DMM with SVVS algorithm
A	1.02 Gbs	0.186 Gbs
B	3.5 Gbs	1.65 Gbs
C	15 Gbs	4.5 Gbs
D	Over 128 Gbs	45 Gbs

### The SVVS improves the accuracy of the approach

Table 3 compares the number of clusters predicted using the two approaches. Both the SVVS algorithm of the infinite DMM model and the EM algorithm of the finite DMM model obtained the correct numbers of clusters for datasets A and B. However, the number of taxonomic units was significantly larger in datasets C and D, and the SVVS approach achieved the most accurate predictions. Moreover, Table 4 compares the ARI values of the two methods. The SVVS algorithm of the infinite DMM model demonstrated a better performance than the conventional finite DMM model for all real datasets. Specifically, SVVS showed the highest ARI value (ARI = 0.98) for dataset A; coversely, the ARI value of the finite DMM with the EM algorithm was 0.76. For dataset B, the ARI values were slightly reduced in the performance of the SVVS (ARI = 0.66) and EM algorithms (ARI = 0.44). The number of taxonomic units in dataset B (3347) was significantly larger than that in dataset A (888). For dataset C, the number of taxonomic units (10,119) was considerably larger than that in datasets A and B; however, the number of samples (162) was smaller than that in datasets A (393) and B (336). Thus, we observed the lowest ARI values across datasets for the SVVS (ARI = 0.48) and EM algorithms (ARI = 0.21). Although dataset D had the largest number of taxonomic units (55,964), the sample size was large (1081). The ARI value of the SVVS approach in dataset D (ARI = 0.5) was better than that in dataset C. The EM algorithm of the finite DMM model was not able to complete its estimation in dataset D, in which the dimensionality of the microbial data was the highest.

**Table 3:** Numbers of clusters predicted by the two approaches for the empirical datasets

Dataset	True numbers of clusters	Predicted numbers of clusters	
		Finite DMM with EM algorithm	Infinite DMM with SVVS algorithm
A	2	2	2
B	3	3	3
C	2	3	2
D	2	Failed	2

**Table 4:** ARI scores of the two approaches for the empirical datasets

Datasets	Finite DMM with EM algorithm	Infinite DMM with SVVS algorithm
A	0.76	0.98
B	0.44	0.66
C	0.21	0.48
D	Failed	0.5

**Fig. 1:**
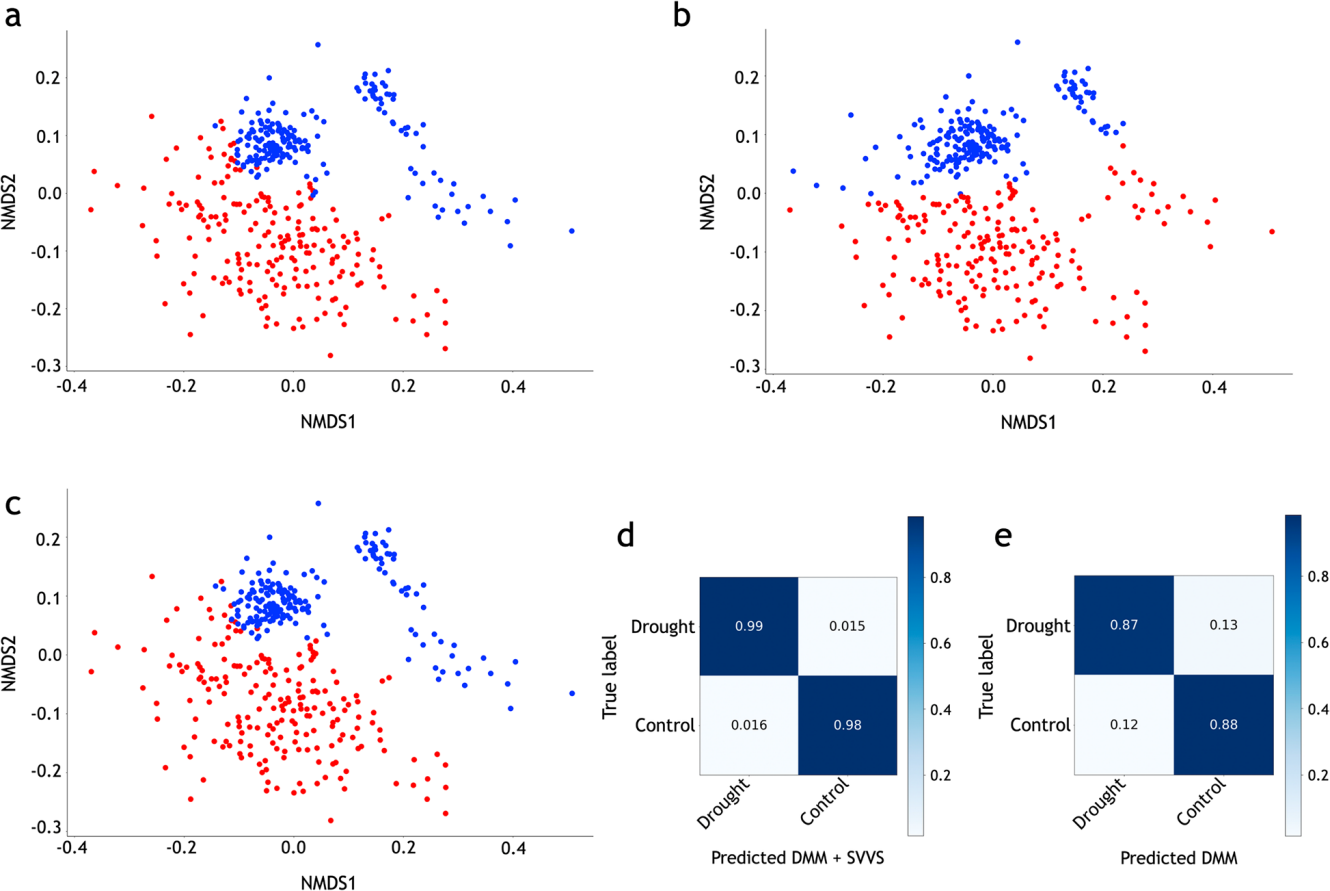
Non-metric multidimensional scaling (NMDS) and confusion matrix plots of dataset A with labels indicating predicted class using the two approaches and true group. **a** Infinite Dirichlet multinomial mixture (DMM) with the stochastic variational variable selection (SVVS) algorithm. **b** The finite DMM with EM algorithm. **c** True labels. Red-colored circles denote the control and blue-colored circles denote drought. **d** Confusion matrix obtained by SVVS. **d** Confusion matrix obtained by previous method

Furthermore, to address graphical visualizations for the cluster labels that were predicted by the SVVS approach for the dataset A, we used non-metric multidimensional scaling (NMDS), which was performed on the unweighted UniFrac distance, to generate two-dimensional positions for community samples. Figure 1a and b show that the two groups of dataset A are separated by both approaches. Figure 1c shows the true label of dataset A. The confusion matrix plots for dataset A calculated by the SVVS and previous method are shown in Figs. 1d and e. Figure S1a-d show the estimated values of the mixing coefficients $\pi _{k}$ in datasets A, B, C, and D after convergence. Evidently, there are some clusters in which their estimated mixing coefficients are close to zero after convergence. Thus, an accurate number of clusters can be obtained. Figure S1a shows the strongest support for 2 clusters in dataset A because $\pi _{2}$ and $\pi _{4}$ have large values; Fig. S1b shows the highest probability of 3 clusters in dataset B because $\pi _{2}$, $\pi _{3}$ and $\pi _{5}$ have large values; and Fig. S1c and d show the highest probability of 2 clusters in datasets C and D. Figure S2 shows the values of the variational lower bound during the estimation iterations in dataset A. The initial number of clusters was 10. Figure S2 shows that the number of clusters decreases rapidly with a significant increase in the variational lower bound. As the change in the lower variational bound value decreases, the speed of the decrease in the number of clusters slows. When the variational lower bound value converges, the number of clusters in dataset A is 2.

### The selected microbiome species mapped on the phylogenetic trees

**Fig. 2:**
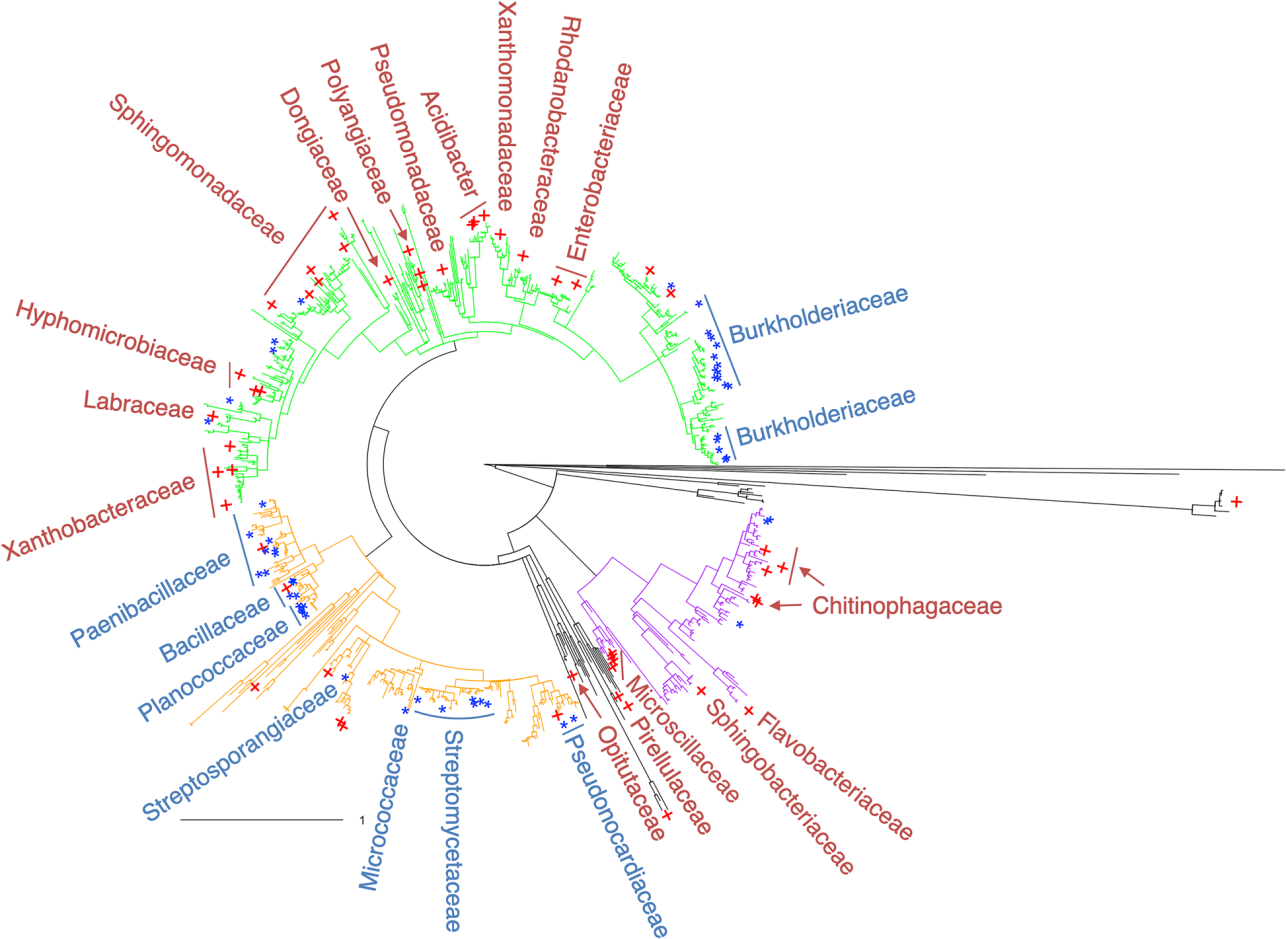
Microbial species selected using the stochastic variational variable selection (SVVS) approach and mapped on the phylogenetic tree for dataset A. Mapping of 100 selected microbiome species. Red-colored plus symbols denote the control and blue-colored stars denote drought. The identification of group-microbiome associations is based on testing for pairwise correlation between allocation variables (drought and control conditions) and the number of counts of top 50 and 100 selected microbiome species using Spearman correlation. Green-colored clade denotes internal node numbers from 12 to 913. Orange-colored clade denotes internal node numbers from 945 to 1448. Purple-colored clade denotes internal node numbers from 1496 to 1737. Black-colored clade denotes the rest of the tree

The other considerable contribution of SVVS is its ability to select a minimum core set of microbial species that shows significant differences among the clusters obtained in the analysis. Specifically, Figs. 2 and S4 show that the top 100 and 50 selected microbiome species in dataset A mapped on the 16S phylogenetic tree. Table S1 shows the average of $\phi _{ij}$ over $i^{th}$ sample that are ordered in decreasing order (from largest to smallest) in dataset A. Figure S3a shows the histogram of the average of $\phi _{ij}$ over $i^{th}$ sample in dataset A. The identification of group-microbiome associations is based on testing for pairwise correlations between allocation variables (drought and control conditions) and the number of counts of the top 50 and 100 selected microbiome species using Spearman correlation. Most microbiome families that were significantly associated with plant growth promotion under drought conditions were observed in the orange region of the tree. Our results are consistent with those of previous studies. For example, many species of bacterial families, including *Micrococcaceae, Paenibacillaceae, Bacillaceae*, and *Planococcaceae*, showed a strong dominance in ecosystems after the impact of wildfires on living organisms [43].

Moreover, Fig. S5 shows the top 100 selected microbiome species in dataset B mapped on the 16S phylogenetic tree. Table S2 shows the average of $\phi _{ij}$ over $i^{th}$ sample that are ordered in decreasing order (from largest to smallest) in dataset B. Figure S3b shows the histogram of the average of $\phi _{ij}$ over $i^{th}$ sample in dataset B. The identification of group-microbiome associations is based on testing for pairwise correlations between allocation variables (CDI cases and non-diarrheal controls) and the number of counts of the top 100 selected microbiome species using Spearman correlation. The green region of the tree includes most microbiome species that show significant associations with non-diarrheal controls. Several dominant species that were significantly associated with CDI were observed in the orange region of the tree. A mixture of the two groups was observed in the purple region. Specifically, numerous microbiome species belonging to the *Bacteroidaceae*, *Porphyromonadaceae*, and *Rikenellaceae* families were observed in the green region of the tree. Several studies have shown that several bacterial species within these families are largely absent in CDI cases and are closely associated with non-diarrheal controls [26, 44]. One of the main risk factors is antibiotic treatments that alter the host nutritional landscape to produce the essential branched-chain amino acids and proline for *C. difficile* growth and to suppress the return of members of the *Rikenellaceae*, *Bacteroidaceae* families [45, 46].

### SVVS improves enterotype clustering

**Fig. 3:**
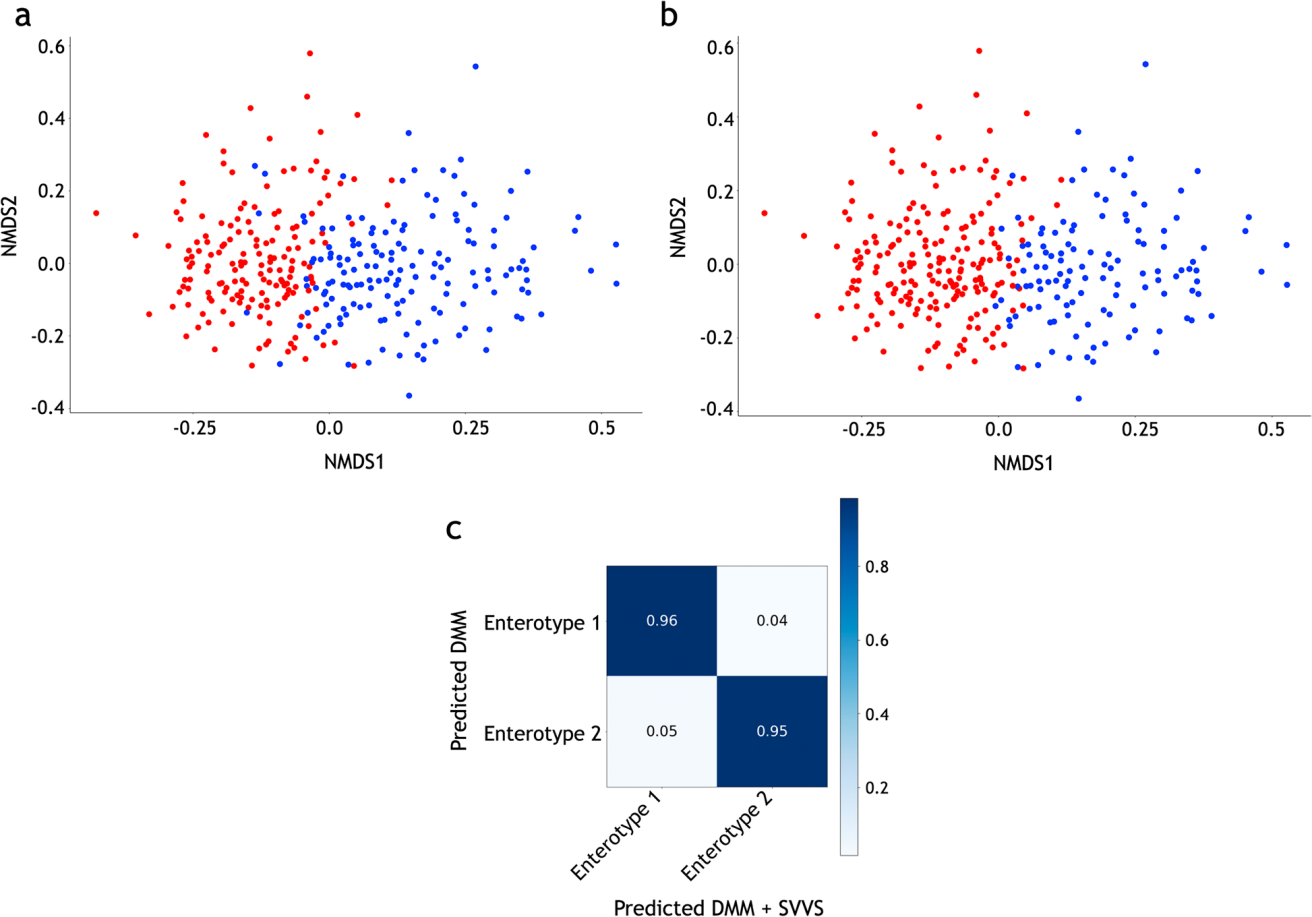
Enterotypes of the healthy human microbiome from the stool samples in dataset E. **a** Labeled enterotypes of the finite Dirichlet multinomial mixture (DMM) with the EM algorithm. **b** Labeled enterotypes of the infinite DMM with the stochastic variational variable selection (SVVS) algorithm. Red color denotes Enterotype 1 and blue color denotes Enterotype 2. **c** Confusion matrix obtained by SVVS and previous method

Figures 3a and b show that the SVVS algorithm of the infinite DMM model and the EM algorithm of the finite DMM model revealed two enterotypes of dataset E. The NMDS plots with the unweighted UniFrac distances showed that two enterotypes were clearly separated by the two approaches. The confusion matrix plot for dataset E calculated by the SVVS and previous method is shown in Fig. 3c. Figure S6 shows the Shannon diversity index was significantly different between the two enterotypes. Moreover, the top 100 microbiome species with the highest average values of $\phi _{ij}$ over $i^{th}$ sample are selected. The identification of enterotype-microbiome associations is based on testing for pairwise correlations between allocation variables and the number of counts of the top 100 selected microbiome species using Spearman correlation. The top 100 selected microbiome species in dataset E, which significantly contributed to the enterotype clustering process, were mapped on the 16S phylogenetic tree. Figure S7 shows that the two enterotypes were clearly separated on the phylogenetic tree. Enterotype 2 had the highest levels of the genus *Bacteroides*. In the previous studies [47–49], the populations such as the European population, which consumes more animal protein and fats, show the dominance of the *Bacteroides* enterotype. Alternatively, Enterotype 1 showed a lower relative abundance of *Bacteroides* than in Enterotype 2 but had higher levels of the genera *Alistipes* and *Parabacteroides* (phylum *Bacteroidetes*), which characterize the *Bacteroides* enterotype. Moreover, the presence of the genera *Roseburia*, *Ruminococcus*,* Faecalibacterium*, *Subdoligranulum*, and *Lachnospiraceae* (phylum *Firmicutes*) was observed in Enterotype 1.

## Discussion

Rapid identification of the minimum core set of taxonomic units in high-dimensional data of microbial studies is essential to further our understanding of microbial community structures in clustering analysis. The intensive concentration of a small number of relevant taxonomic units that significantly contribute to the task of clustering will not only increase the performance of these analyses but also open new opportunities for studies that explore the important associations of microbial communities with human diseases, precision medicine, and environmental conditions.

As the substantial increases in the dimensionality of the microbial datasets cause computational burden and poor performance with previous methods, the proposed approach can satisfy the high demands of the microbiome analysis. Our SVVS approach is useful in several aspects. First, SVVS integrates an indicator variable into the approach of the infinite DMM model to identify significant microbiome species (or taxonomic units) and use SVI to overcome computational limitations. Thus, the SVVS approach quickly identify the core set of microbial species (or taxonomic units), considerably improving the performance of the infinite DMM model. In particular, the SVVS method can complete its main tasks in massive microbiome datasets [27] that the previous methods cannot perform. Moreover, SVVS focuses on identifying the important taxonomic units that vary per sample rather than per cluster. Within a set of samples that are assigned to a cluster, each sample has a different group of important taxonomic units that are selected in the clustering process. For example, some human populations (or clusters) exist, such as the Japanese, American, and European populations. Each individual (or sample) in a population (or cluster) usually has a different group of important microbial species because of differences in daily diet. If we can use other types of data, such as host genotypes and host metabolism, in the future, we will obtain more information about important microbial species that are selected for assigning samples to clusters. Second, a stick-breaking representation is proposed to extend the finite DMM model to an infinite case. This solution treats the total number of clusters as a variational parameter, which can help avoid the disadvantages of determining the number of clusters before running the algorithms. Therefore, SVVS can identify the main enterotypes of the healthy human microbiome and detect the important microbiome species that contribute to the variation of the different community compositions.

This study uses 16S ribosomal RNA genes datasets. SVVS identify a core set of important microbial species (or taxonomic units); however, their taxonomic resolution is limited at the genus level (e.g., *Bifidobacteria*). Applying SVVS to metagenomic count data will provide information on microbial species such as bacteria (e.g., *Bifidobacterium longum*) at strain-level resolution based on shotgun metagenomic sequencing. However, the high dimensionality of metagenomic count data [50, 51] challenges the performance of the SVVS approach. Furthermore, shotgun metagenome sequencing can provide additional information regarding the functional potential of the microbiome. Integration of microbiome functional profiles can improve the performance of clustering algorithms and contribute to the interpretation of host-microbial co-metabolism interactions.

In recent years, several studies have highlighted the substantial role of large-scale analysis in discovering microbiome connections with host metabolism, host genetics in human health, medication [52, 53], and agroecosystems [54]. An increasing number of multi-omics datasets have been published, such as the integration of metagenomics, metatranscriptomics, metaproteomics [55], whole-genome sequencing, and whole-transcriptome sequencing of the TCGA cancer microbiome [56]. In the future, we plan to extend the SVVS approach to a comprehensive analysis of multi-omics datasets. The main approach of the SVVS can be developed for the other Bayesian mixture models such as beta-mixture models for microarray gene expression datasets [57], and multinomial mixture model for ChIP-exo sequencing data [58]. Therefore, this approach provides to new opportunities for discovering the significant associations of microbes with specific nutrients and medication or the important interactions between plants, microbes, and soils.

## Conclusion

In conclusion, the proposed stochastic variational variable selection approach can significantly improve the performance of the Dirichlet multinomial mixture model for analyzing high-dimensional microbial data sets. The selected minimum core set of microbial species facilitates the detection of features that contribute most to the differences between samples. This study will contribute to and stimulate ongoing efforts to improve the performance of metagenomic statistical models that rapidly identify the key species of the environmental and human microbiomes in multiple fields of study, including the industrial sectors, and health and medical field.

## Supplementary Information

**Additional file 1.** Supplemental Materials and Methods.

**Additional file 2:** **Figure S1.** The estimated values of the mixing coefficients. a. dataset A; b. dataset B; c. dataset C; d. dataset D. **Figure S2.** Variational lower bound function values obtained by SVVS during iterations for dataset A. We take the setting of an initial number of the clusters as 10, as an example. The numbers on top of the vertical dashed line are the present number of clusters at the current iteration. **Figure S3.** Histogram of the average of $\phi_{ij}$. The dashed line is a bound to select microbiome species. a. dataset A; b. dataset B. **Figure S4.** Microbial species selected using the stochastic variational variable selection (SVVS) approach and mapped on the phylogenetic tree for dataset B (*Clostridium difficile* infection (CDI) disease). Red-colored plus symbols denote for CDI cases and blue-colored stars denote non-diarrheal control. **Figure S5.** **a.** Alpha diversity of two enterotypes labeled by SVVS (Circle symbols denote Enterotype 1 and triangle symbols denote Enterotype 2); **b.** Top 100 important microbiome species that selected using the SVVS approach. Red color denotes Enterotype 1 and blue color denotes Enterotype 2.

**Additional file 3:** **Table S1.** The probability of microbiome species that are selected by SVVS in dataset A. The values are ordered in descending order (from largest to smallest). **Table S2.** The probability of microbiome species that are selected by SVVS in dataset B. The values are ordered in descending order (from largest to smallest).

## Data Availability

https://github.com/tungtokyo1108/SVVS

## Abbreviations

ARI: Adjusted Rand index
CDI: Clostridium difficile infection
DMM: Dirichlet multinomial mixture
EM: Expectation maximization
ELBO: Evidence lower bound
IBD: Inflammatory bowel disease
KL: Kullback-Leibler
MCMC: Markov chain Monte Carlo
OB: Obesity
QIIME: Quantitative Insights Into Microbial Ecology
OTUs: Operational taxonomic units
RI: Rand index
SVI: Stochastic variational inference
SVVS: Stochastic variational variable selection
