# Supplementary material for: Stochastic variational variable selection for high-dimensional microbiome data
Source: Microbiome. 2022 Dec 24;10:236. doi: 10.1186/s40168-022-01439-0 (PMC9789572; doi:10.1186/s40168-022-01439-0)
Supplement: Supplementary file 2 — Additional file 1. Supplemental Materials and Methods. [file 40168_2022_1439_MOESM1_ESM.pdf]

Stochastic variational variable selection for high-dimensional microbiome data

Tung Dang,[1]*, Kie Kumaishi*[2]*, Erika Usui*[2]*, Shungo Kobori*[2]*, Takumi Sato*[2]*, Yusuke Toda*[1]*, Yuji Yamasaki*[3]*, Hisashi Tsujimoto*[3]*, Yasunori Ichihashi*[2] *and Hiroyoshi Iwata*[*,1]

[1] Graduate School of Agricultural and Life Sciences, The University of Tokyo, Bunkyo, Tokyo, 113-8657, Japan

[2] RIKEN BioResource Research Center, Tsukuba, Ibaraki 305-0074, Japan

[3] Arid Land Research Center, Tottori University, Tottori, 680-0001, Japan

[*] Correspondence: hiroiwata@g.ecc.u-tokyo.ac.jp

1 Mean-Field Variational Inference for DMM Model

We expand specifically the variational lower bound equation as follows:

$$
\begin{aligned}
\mathcal{L} = & \sum_{k=1}^{K_{max}} \mathrm{E}_q \left[\log\left(p\left(\gamma_k|1,\kappa\right)\right)\right] - \sum_{k=1}^{K_{\max}} \mathrm{E}_q\left[\log\left(q\left(\gamma_k|\vartheta_k,\vartheta_k^{'}\right)\right)\right] \\
& + \sum_{i=1}^{N}\sum_{k=1}^{K_{max}} \mathrm{E}_q\left[\log p(Z_i^k|\gamma_1,\gamma_2,...,\gamma_{K_{max}})\right] - \sum_{i=1}^{N}\sum_{k=1}^{K_{\max}} \mathrm{E}_q\left[\log\left(q\left(Z_i^k|r_i^k\right)\right)\right] \\
& + \sum_{i=1}^{N}\sum_{j=1}^{S} \mathrm{E}_q\left[\log p(\phi_{ij}|\epsilon_{j_1},\epsilon_{j_2})\right] - \sum_{i=1}^{N}\sum_{j=1}^{S} \mathrm{E}_q\left[\log\left(q\left(\phi_{ij}|f_{ij}\right)\right)\right] \\
& + \sum_{j}^{S} \mathrm{E}_q\left[\log\left(p\left(\epsilon_j|\xi\right)\right)\right] - \sum_{j}^{S} \mathrm{E}_q\left[\log\left(q\left(\epsilon_j|\xi^*\right)\right)\right] \\
& + \sum_{j=1}^{S}\sum_{k=1}^{K_{max}} \mathrm{E}_q\left[\log\left(p\left(\alpha_{kj}|\lambda_{kj}\right)\right)\right] - \sum_{j=1}^{S}\sum_{k=1}^{K_{max}} \mathrm{E}_q\left[\log\left(q\left(\alpha_{kj}|\lambda_{kj}^*\right)\right)\right] \\
& + \sum_{j=1}^{S} \mathrm{E}_q\left[\log\left(p\left(\beta_j|\iota_j\right)\right)\right] - \sum_{j=1}^{S} \mathrm{E}_q\left[\log\left(q\left(\beta_j|\iota_j^*\right)\right)\right]
\end{aligned}
\tag{1}
$$

To compute the variational expectations $E_q[.]$ in equation (1), we use the properties of exponential family distribution. If variational distributions for $q(\alpha_{kj}|\lambda_{kj})$ and $q(\beta_j|\iota_j)$ are Dirichlet distributions, then the exponential family representations are given by

$$
\begin{aligned}
q\left(\boldsymbol{\alpha}_k|\boldsymbol{\lambda}_k\right) &= \exp\left[\left(\sum_{j=1}^{S}\left(\lambda_j^k-1\right)\log\left(\alpha_j^k\right)\right) + \log\Gamma\left(\sum_{j=1}^{S}\lambda_j^k\right) - \sum_{j=1}^{S}\left(\log\Gamma\left(\lambda_j^k\right)\right)\right] \\
q\left(\boldsymbol{\beta}|\boldsymbol{\iota}\right) &= \exp\left[\left(\sum_{j=1}^{S}\left(\iota_j-1\right)\log\left(\beta_j\right)\right) + \log\Gamma\left(\sum_{j=1}^{S}\iota_j\right) - \sum_{j=1}^{S}\left(\log\Gamma\left(\iota_j\right)\right)\right]
\end{aligned}
$$

So the natural parameters and sufficient statistics of the Dirichlet distributions for α and β are $\eta_{\alpha_{kj}} = \lambda_{kj} - 1$, $T(\alpha_{kj}) = \log(\alpha_{kj})$ and $\eta_{\beta_j} = \iota_j - 1$, $T(\beta_j) = \log(\beta_j)$, resepctively. The approximation expectations are

$$
\begin{aligned}
\mathrm{E}\left[\alpha_{kj}\right] &= \frac{\lambda_{kj}}{\sum_{j'=1}^{S}\lambda_{kj'}} & \mathrm{E}\left[\beta_j\right] &= \frac{\iota_j}{\sum_{j'=1}^{S}\iota_{j'}} \\
\mathrm{E}\left[\log\left(\alpha_{kj}\right)\right] &= \psi\left(\lambda_{kj}\right) - \psi\left(\sum_{j'=1}^{S}\lambda_{kj'}\right) & \mathrm{E}\left[\log\left(\beta_j\right)\right] &= \psi\left(\iota_j\right) - \psi\left(\sum_{j'=1}^{S}\iota_{j'}\right)
\end{aligned}
$$

where $\psi(.)$ is the digamma function.

With truncated stick-breaking representation of Dirichlet Mixture process, variational factors for the stick lengths $q\left(\gamma_k|\vartheta_k,\vartheta_k^{'}\right)$ are Beta distributions and the assignment variable $Z_i^k = \mathrm{I}\left[Z_i = k\right]$ for the ith sample allocation is governed by a multinomial distribution indexed by a variational parameter r_{ik}. The computation of the approximate expectation for the parameters of truncated stick-breaking representation have been considered

carefully by Blei and Jordan (2006). The results of variational expectation for both variables are as follows:

$$q\left(Z_i = k\right) = r_i^k$$
$$q\left(Z_i > k\right) = \sum_{k'=k+1}^{K_{\max}} r_i^{k'}$$
$$\mathrm{E}_q\left[\log\left(\gamma_k\right)\right] = \psi\left(\vartheta_k\right) - \psi\left(\vartheta_k + \vartheta_k^{'}\right)$$
$$\mathrm{E}_q\left[\log\left(1-\gamma_k\right)\right] = \psi\left(\vartheta_k^{'}\right) - \psi\left(\vartheta_k + \vartheta_k^{'}\right)$$

2 Stochastic Optimization of the Variational Parameters

2.1 Compute the variational function parameters for the position of sample i to be reallocated to cluster k

Following the principles of the variational inference (Bishop. 2006, Blei and Jordan. 2006, Hoffman 2013), variational parameters of Z_i^k is the local parameters, we consider the derivation of the update equation for only one variable by fixing the others' distribution. The optimal coordinate updates are derived for local parameters. The log of the optimized factor to the posterior distribution of Z_i^k is

$$\log Q^*\left(Z\right) = E_{\phi,\gamma,\epsilon,\alpha,\beta}\left[\log p\left(Z,\phi,\gamma,\epsilon,\alpha,\beta\right)\right] + const = \sum_{i=1}^{N}\sum_{k=1}^{K} Z_i^k \log\left(r_i^k\right) + const$$

$$\begin{aligned}\log r_i^k = {} & \textstyle\sum_{j=1}^{S} \mathrm{E}_Q\left[\phi_{ij}\right]\left(\mathrm{E}_Q\left[log\left(\frac{\Gamma\left(\sum_{j=1}^{S}\alpha_{kj}\right)}{\Gamma\left(\sum_{j=1}^{S}X_{ij}+\sum_{j=1}^{S}\alpha_{kj}\right)}\right)\right] + \sum_{j=1}^{S}\mathrm{E}_Q\left[log\left(\frac{\Gamma(X_{ij}+\alpha_{kj})}{\Gamma(\alpha_{kj})}\right)\right] + log(J_i!) + \sum_{j=1}^{S} log\left(\frac{1}{X_{ij}!}\right)\right) \\ & +\mathrm{E}_Q\left[log(\lambda_k)\right] + \textstyle\sum_{k'=1}^{k-1}\mathrm{E}_Q\left[log(1-\lambda_{k'})\right]\end{aligned} \tag{2}$$

$$\mathrm{E}_Q\left[Z_i^k\right] = r_i^k = \exp\left\{\log\left(r_i^k\right)\right\}$$

The expected logarithm of the functions $\mathrm{E}_Q\left[log\left(\frac{\Gamma\left(\sum_{j=1}^{S}\alpha_{kj}\right)}{\Gamma\left(\sum_{j=1}^{S}X_{ij}+\sum_{j=1}^{S}\alpha_{kj}\right)}\right)\right]$ and $\mathrm{E}_Q\left[log\left(\frac{\Gamma(X_{ij}+\alpha_{kj})}{\Gamma(\alpha_{kj})}\right)\right]$ in equation (2) have not the closed form. Thus, the calculation of these equations are analytically intractable. To use standard form of the variational inference (Bishop. 2006) or the stochastic variational inference (Hoffman et al. 2013), we need a closed-form expression. In order to overcome this problem, we consider a common approach:

- By applying a first-order Taylor expansion to preserve a bound, intractable expectations are avoided. By using these results, the variational parameters are updated as follow:

$$\begin{aligned} & log\left(\frac{\Gamma(X_{ij}+\alpha_{kj})}{\Gamma(\alpha_{kj})}\right) \geq log\left(\frac{\Gamma(X_{ij}+\overline{\alpha_{kj}})}{\Gamma(\overline{\alpha_{kj}})}\right) + \frac{\partial F(\alpha_{kj})}{\partial\alpha_{kj}}\frac{\partial\alpha_{kj}}{\partial log(\alpha_{kj})}\Big|_{\alpha_{kj}=\overline{\alpha_{kj}}}\left(log(\alpha_{kj}) - log(\overline{\alpha_{kj}})\right) \\ & \mathrm{E}_Q\left[log\left(\frac{\Gamma(X_{ij}+\alpha_{kj})}{\Gamma(\alpha_{kj})}\right)\right] \geq log\left(\frac{\Gamma(X_{ij}+\overline{\alpha_{kj}})}{\Gamma(\overline{\alpha_{kj}})}\right) + \overline{\alpha_{kj}}\left[\Psi\left(\overline{\alpha_{kj}}+X_{ij}\right) - \Psi\left(\overline{\alpha_{kj}}\right)\right]\left(\mathrm{E}_Q\left[log(\alpha_{kj})\right] - log(\overline{\alpha_{kj}})\right) \\ & \geq log\left(\frac{\Gamma(X_{ij}+\overline{\alpha_{kj}})}{\Gamma(\overline{\alpha_{kj}})}\right) + \overline{\alpha_{kj}}\left[\Psi\left(\overline{\alpha_{kj}}+X_{ij}\right) - \Psi\left(\overline{\alpha_{kj}}\right)\right]\left(\Psi(\lambda_{kj}^*) - \Psi\left(\textstyle\sum_{j=1}^{S}\lambda_{kj}^*\right) - log(\overline{\alpha_{kj}})\right)\end{aligned}$$

$$\begin{aligned} & \mathrm{E}_Q\left[log\left(\frac{\Gamma\left(\sum_{j=1}^{S}\alpha_{kj}\right)}{\Gamma\left(\sum_{j=1}^{S}X_{ij}+\sum_{j=1}^{S}\alpha_{kj}\right)}\right)\right] \geq log\left(\frac{\Gamma\left(\sum_{j=1}^{S}\overline{\alpha_{kj}}\right)}{\Gamma\left(\sum_{j=1}^{S}X_{ij}+\sum_{j=1}^{S}\overline{\alpha_{kj''}}\right)}\right) \\ & +\textstyle\sum_{j=1}^{S}\left[\Psi\left(\sum_{j=1}^{S}\overline{\alpha_{kj}}\right) - \Psi\left(\sum_{j=1}^{S}X_{ij} + \sum_{j=1}^{S}\overline{\alpha_{kj}}\right)\right]\overline{\alpha_{kj}}\left(\Psi(\lambda_{kj}^*) - \Psi\left(\sum_{j=1}^{S}\lambda_{kj}^*\right) - log(\overline{\alpha_{kj}})\right)\end{aligned}$$

Based on this framework, the variational parameters of Z_{ik} are updated as follow:

$$\begin{aligned}\log r_i^k = {} & \textstyle\sum_{j=1}^{S} f_{ij} log\left(\frac{\Gamma\left(\sum_{j=1}^{S}\overline{\alpha_{kj}}\right)}{\Gamma\left(\sum_{j=1}^{S}X_{ij}+\sum_{j=1}^{S}\overline{\alpha_{kj''}}\right)}\right) \\ & +\textstyle\sum_{j=1}^{S} f_{ij}\left[\Psi\left(\sum_{j=1}^{S}\overline{\alpha_{kj}}\right) - \Psi\left(\sum_{j=1}^{S}X_{ij}+\sum_{j=1}^{S}\overline{\alpha_{kj}}\right)\right]\overline{\alpha_{kj}}\left(\Psi(\lambda_{kj}^*) - \Psi\left(\sum_{j=1}^{S}\lambda_{kj}^*\right) - log(\overline{\alpha_{kj}})\right) \\ & +\textstyle\sum_{j=1}^{S} f_{ij}\left[log\left(\frac{\Gamma(X_{ij}+\overline{\alpha_{kj}})}{\Gamma(\overline{\alpha_{kj}})}\right) + \overline{\alpha_{kj}}\left[\Psi\left(\overline{\alpha_{kj}}+X_{ij}\right) - \Psi\left(\overline{\alpha_{kj}}\right)\right]\left(\Psi(\lambda_{kj}^*) - \Psi\left(\sum_{j=1}^{S}\lambda_{kj}^*\right) - log(\overline{\alpha_{kj}})\right)\right] \\ & +\Psi(\vartheta_k) - \Psi(\vartheta_k + \vartheta_k^{'}) + \textstyle\sum_{k'=1}^{k-1}\Psi(\vartheta_{k'}^{'}) - \Psi(\vartheta_{k'} + \vartheta_{k'}^{'})\end{aligned} \tag{3}$$

2.2 Compute the variational function parameters of indicator variables for microbiome selection

Similarity, the optimized solution to the posterior distribution of variable selection ϕ_{ij}

$$\log Q^*(\phi) = E_{Z,\gamma,\epsilon,\alpha,\beta}\left[\log p(Z,\phi,\gamma,\epsilon,\alpha,\beta)\right] + const = \sum_{i=1}^{N}\sum_{j=1}^{S}\phi_{ij}\log(f_{ij}) + const$$

$$\begin{aligned}\log f_{ij}^{\phi_{ij}} &= \textstyle\sum_{k=1}^{K_{max}} r_{ik} log\left(\frac{\Gamma(\sum_{j=1}^{S}\overline{\alpha_{kj}})}{\Gamma(\sum_{j=1}^{S}X_{ij}+\sum_{j=1}^{S}\overline{\alpha_{kj''}})}\right)\\ &+\textstyle\sum_{k=1}^{K_{max}} r_{ik}\left[\Psi\left(\sum_{j=1}^{S}\overline{\alpha_{kj}}\right)-\Psi\left(\sum_{j=1}^{S}X_{ij}+\sum_{j=1}^{S}\overline{\alpha_{kj}}\right)\right]\overline{\alpha_{kj}}\left(\Psi(\lambda_{kj}^*)-\Psi\left(\sum_{j=1}^{S}\lambda_{kj}^*\right)-log(\overline{\alpha_{kj}})\right)\\ &+\textstyle\sum_{k=1}^{K_{max}} r_{ik}\left[log\left(\frac{\Gamma(X_{ij}+\overline{\alpha_{kj}})}{\Gamma(\overline{\alpha_{kj}})}\right)+\overline{\alpha_{kj}}\left[\Psi\left(\overline{\alpha_{kj}}+X_{ij}\right)-\Psi\left(\overline{\alpha_{kj}}\right)\right]\left(\Psi(\lambda_{kj}^*)-\Psi\left(\sum_{j=1}^{S}\lambda_{kj}^*\right)-log(\overline{\alpha_{kj}})\right)\right]\\ &+[\Psi(\xi_1^*)-\Psi(\xi_1^*+\xi_2^*)]\end{aligned} \quad (4)$$

$$\begin{aligned}\log f_{ij}^{1-\phi_{ij}} &= log\left(\frac{\Gamma(\sum_{j=1}^{S}\overline{\beta_j})}{\Gamma(\sum_{j=1}^{S}X_{ij}+\sum_{j=1}^{S}\overline{\beta_{j''}})}\right)\\ &+\textstyle\sum_{j=1}^{S}\left[\Psi\left(\sum_{j=1}^{S}\overline{\beta_j}\right)-\Psi\left(\sum_{j=1}^{S}X_{ij}+\sum_{j=1}^{S}\overline{\beta_j}\right)\right]\overline{\beta_j}\left(\Psi(\iota_j^*)-\Psi\left(\sum_{j=1}^{S}\iota_j^*\right)-log(\overline{\beta_j})\right)\\ &+log\left(\frac{\Gamma(X_{ij}+\overline{\beta_j})}{\Gamma(\overline{\beta_j})}\right)+\overline{\beta_j}\left[\Psi\left(\overline{\beta_j}+X_{ij}\right)-\Psi\left(\overline{\beta_j}\right)\right]\left(\Psi(\iota_j^*)-\Psi\left(\sum_{j=1}^{S}\iota_j^*\right)-log(\overline{\alpha_{kj}})\right)\\ &+[\Psi(\xi_2^*)-\Psi(\xi_1^*+\xi_2^*)]\end{aligned} \quad (5)$$

2.3 Updating variational parameters of α

Based on the principal framework of the stochastic variational inference (Hoffman et al. 2013), we consider parameters of α as global parameters. These parameters are updated by a stochastic gradient step and noisy estimations of the natural gradient of the variational objective with respect to α_{kj}. Following the computational method of the natural gradient (Hoffman et al. 2013), we compute the natural gradient of equation (1) with respect to the global variational parameters of α_{kj}.
We consider Dirichlet distribution as a prior distribution with concentration parameters λ_{kj}, so a conditional distribution of profile given the observation data has form of the Dirichlet distribution. We consider the representation of the exponential family,

$$p(\alpha|Z,\phi,\gamma,\epsilon,\beta) = h(\alpha)\exp\left(\eta(Z,\phi,\gamma,\epsilon,\beta)^T t(\alpha) - a(\eta(Z,\phi,\gamma,\epsilon,\beta))\right)$$

where: $h(.)$ is the base measure; $a(.)$ is the log-normalize; $\eta(.)$ is the natural parameter; $t(.)$ is the sufficient statistics.

As above assumption of the variational parameters, we set $q(\alpha|\lambda^*)$ to be Dirichlet distribution as the complete conditional distributions. So we get

$$q(\alpha|\lambda^*) = h(\alpha)\exp\left((\lambda^*)^T t(\pi) - a(\lambda^*)\right)$$

We consider the lower bound for only α,

$$\begin{aligned}\mathcal{L}(\alpha) &= \mathrm{E}_Q\left[\log p(\alpha|Z,\phi,\gamma,\epsilon,\beta)\right] - \mathrm{E}_Q\left[q(\alpha|\lambda^*)\right]\\ &= \mathrm{E}_Q\left[\log\{h(\alpha)\} + \eta(Z,\phi,\gamma,\epsilon,\beta)^T t(\alpha) - a(\eta(Z,\phi,\gamma,\epsilon,\beta)) - \log\{h(\alpha)\} - (\lambda^*)^T t(\alpha) + a(\lambda^*)\right]\\ &= \mathrm{E}_Q\left[\eta(Z,\phi,\gamma,\epsilon,\beta)^T t(\alpha) - a(\eta(Z,\phi,\gamma,\epsilon,\beta)) - (\lambda^*)^T t(\alpha) + a(\lambda^*)\right]\\ &= \mathrm{E}_Q\left[\eta(Z,\phi,\gamma,\epsilon,\beta)\right]^T\left[\nabla_{\lambda^*}\{a(\lambda^*)\}\right] - a(\eta(Z,\phi,\gamma,\epsilon,\beta)) - (\lambda^*)^T\left[\nabla_{\lambda^*}\{a(\lambda^*)\}\right] + a(\lambda^*)\end{aligned}$$

where, the expected value of the sufficient statistics is the gradient of log normalizer $\mathrm{E}_Q[t(\alpha)] = \nabla_{\lambda^*}\{a(\lambda^*)\}$
If the classical principal of the gradient method for maximization is used directly to find a maximum of $\mathcal{L}(\alpha)$ based on taking step of size ρ in direction of the gradient, the optimized results is

$$\lambda_{(t+1)}^* = \lambda_{(t)}^* + \rho\nabla_{(\lambda^*)}L(\alpha) = \lambda_{(t)}^* + \rho^{(t)}\left[\nabla_{(\lambda^*)}^2\{a(\lambda^*)\}\right]\left\{E_Q\left[\eta(Z,\phi,\gamma,\epsilon,\beta)\right]^T - (\lambda^*)^T\right\}$$

Following (Hoffman et al. 2013), by premultiplying the gradient by the inverse Fisher information $\mathrm{G}(\lambda^*)$ and apply the stochastic natural gradient of the variational objective with respect to α, we get

$$G(\lambda^*) = E_{\lambda^*}\left[\left(\nabla_{(\lambda^*)}\log q(\alpha|\lambda^*)\right)\left(\nabla_{(\lambda^*)}\log q(\alpha|\lambda^*)\right)^T\right] = \nabla_{(\lambda^*)}^2\{a(\lambda^*)\}$$

$$\widehat{\nabla_{(\lambda^*)}}L(\alpha) = \{G(\lambda^*)\}^{-1}\nabla_{(\lambda^*)}L(\alpha) = \{E_Q[\eta(Z,\phi,\gamma,\epsilon,\beta)] - \lambda^*\}$$

$$\begin{aligned}&\lambda^*_{(t+1)} = \lambda^*_{(t)} + \rho \widehat{\nabla_{(\lambda^*)}} L(\alpha) = \lambda^*_{(t)} + \rho \left\{ E_Q \left[\eta \left(Z, \phi, \gamma, \epsilon, \beta \right) \right] - \lambda^*_{(t)} \right\} \\ &= (1-\rho)\, \lambda^*_{(t)} + \rho \left\{ E_Q \left[\eta \left(Z, \phi, \gamma, \epsilon, \beta \right) \right] \right\}\end{aligned} \tag{6}$$

Based traditional variational inference, we get the conditional distribution of α

$$\log p\left(\alpha | Z, \phi, \gamma, \epsilon, \beta\right) = \sum_{i=1}^{N} \sum_{j=1}^{S} \sum_{k=1}^{K_{max}} \log \left(\frac{\Gamma(X_{ij}+\alpha_{kj})}{\Gamma(\alpha_{kj})} \right) \times \log \left(\frac{\Gamma(X_{ij}+\alpha_{kj})}{\Gamma(\alpha_{kj})} \right) \times \mathrm{E}_Q \left[Z_{ik} \right] \mathrm{E}_Q \left[\phi_{ij} \right] + \log \left(\alpha_{kj} \right) \left(\lambda_{kj} - 1 \right) + const$$

$$\mathrm{E}_q \left[\eta \left(Z, \phi, \gamma, \epsilon, \beta \right) \right] = \sum_{i=1}^{N} \mathrm{E}_Q \left[z_{ik} \right] \mathrm{E}_Q \left[\phi_{ij} \right] \overline{\alpha_{kj}} \left[\Psi \left(\sum_{j=1}^{S} \overline{\alpha_{kj}} \right) - \Psi \left(\sum_{j=1}^{S} X_{ij} + \sum_{j=1}^{S} \overline{\alpha_{kj}} \right) + \Psi \left(\overline{\alpha_{kj}} + X_{ij} \right) - \Psi \left(\overline{\alpha_{kj}} \right) \right] \tag{7}$$

By substituting equation (7) into equation (6), the final result of updated variational parameters is

$$\begin{aligned}&\left(\lambda^*_{kj}\right)^{(t+1)} = \left(1 - \rho^{(t)}\right) \left(\lambda^*_{kj}\right)^{(t)} \\ &+\rho^{(t)} \left\{ \lambda_{kj} + \textstyle\sum_{i=1}^{N} r_{ik} f_{ij} \overline{\alpha_{kj}} \left[\Psi \left(\sum_{j=1}^{S} \overline{\alpha_{kj}} \right) - \Psi \left(\sum_{j=1}^{S} X_{ij} + \sum_{j=1}^{S} \overline{\alpha_{kj}} \right) + \Psi \left(\overline{\alpha_{kj}} + X_{ij} \right) - \Psi \left(\overline{\alpha_{kj}} \right) \right] \right\}\end{aligned}$$

2.4 Updating variational parameters of β

Similarity, the stochastic natural gradient of the variational objective with respect to β

$$\begin{aligned}&\left(\iota^*_j\right)^{(t+1)} = \left(1 - \rho^{(t)}\right) \left(\iota^*_j\right)^{(t)} \\ &+\rho^{(t)} \left\{ \iota_j + \textstyle\sum_{i=1}^{N} \left[1 - f_{ij}\right] \overline{\beta_j} \left(\left[\Psi \left(\sum_{j=1}^{S} \overline{\beta_j} \right) - \Psi \left(\sum_{j=1}^{S} X_{ij} + \sum_{j=1}^{S} \overline{\beta_j} \right) \right] + \left[\Psi \left(\overline{\beta_j} + X_{ij} \right) - \Psi \left(\overline{\beta_j} \right) \right] \right) \right\}\end{aligned}$$

2.5 Updating stick-breaking representation

Similarity, the optimized solution to the posterior distribution of unit length sticks γ_k

$$\begin{aligned}&\log Q^* \left(\gamma_k\right) = (1-1) \log \left(\gamma_k\right) + (\nu - 1) \log \left(1 - \gamma_k\right) \\ &+ \sum_{i=1}^{N} \mathrm{E}_Q \left[z_{ik} \right] \log \left(\gamma_k\right) + \sum_{i=1}^{N} \sum_{k'=k+1}^{K_{\max}} E_Q \left[z_{ik'} \right] \log \left(1 - \gamma_k\right) + const\end{aligned} \tag{8}$$

which has the logarithmic form of the Beta distribution and the corresponding variational distributions of breaking proportions $q\left(\gamma_k\right)$ are considered to be the Beta distribution. Then, the standard conditions are satisfied for a closed form coordinate update for local parameters. Based on the stochastic natural gradient, the variational parameters of γ_k are updated by computing variational expectations in equation (8) (Blei and Jordan. 2006, Hoffman et al. 2013),

$$\begin{aligned}&\left(\vartheta_k\right)^{(t+1)} = \left(1 - \rho^{(t)}\right) \left(\vartheta_k\right)^{(t)} + \rho^{(t)} \left\{ 1 + \sum_{i=1}^{N} r_{ik} \right\} \\ &\left(\vartheta'_k\right)^{(t+1)} = \left(1 - \rho^{(t)}\right) \left(\vartheta'_k\right)^{(t)} + \rho^{(t)} \left\{ \nu + \sum_{i=1}^{N} \sum_{k'=k+1}^{K_{\max}} r_{ik'} \right\}\end{aligned}$$

2.6 Updating variational parameters of ϵ

Similarity, the stochastic natural gradient of the variational objective with respect to ϵ

$$\begin{aligned}&\left(\xi^*_{j1}\right)^{(t+1)} = \left(1 - \rho^{(t)}\right) \left(\xi^*_{j1}\right)^{(t)} + \rho^{(t)} \left\{ \xi_{j1} + \textstyle\sum_{i=1}^{N} f_{ij} \right\} \\ &\left(\xi^*_{j2}\right)^{(t+1)} = \left(1 - \rho^{(t)}\right) \left(\xi^*_{j2}\right)^{(t)} + \rho^{(t)} \left\{ \xi_{j2} + \textstyle\sum_{i=1}^{N} \left(1 - f_{ij}\right) \right\}\end{aligned}$$

3 Database description

Tottori, Japan study

Field experiment

A total of 198 soybean accessions registered in the National Agriculture and Food Research Organization Genebank (https://www.gene.affrc.go.jp/) were used. The field trial was conducted in 2018 in an experimental field with sandy soil at the Arid Land Research Center, Tottori University (35°32' N, 134°12' E, 14 m above sea level). Each plot consisted of four plants, and the distances between two rows, two plots, and two individuals were 50, 80, and 20 cm, respectively. Sowing was performed at the beginning of July, followed by thinning after two weeks. Fertilizer (15, 6.0, 20, 11, and 7.0 g m-2 of N, P, K, Mg, and Ca, respectively) was applied to the field before sowing. White mulching sheets (Dupont, Wilmington, DE, USA) were laid to prevent rainwater infiltration and to control soil conditions with artificial irrigation. Watering tubes were installed under the sheets to irrigate the fields. Two watering treatments, non-watered treatment and well-watered treatments, were used to evaluate the influence of drought and control conditions. The watering treatment started after thinning every year, two weeks after sowing. Artificial irrigation was applied for 5 h daily (7:00–9:00, 12:00–14:00, and 16:00–17:00).

DNA extraction

DNA extraction was performed according to our custom protocol using magnetic beads (Kumaishi et al. 2021). Briefly, the collected root tissues were ground to a fine powder using a multi-beads shocker (MB2200(S); Yasui Kikai Co. Osaka, Japan). For each of the collected tissue samples, 500 mg of the powdered sample was transferred into a 1.5 mL tube cooled by liquid nitrogen. Then, 1 mL of lysate binding buffer (1 M LiCl, Cat. #L7026-500ML, Sigma-Aldrich, St. Louis, MO, USA; 100 mM Tris-HCl, Cat. #318-90225, Wako Pure Chemical Corporation, OSA, Japan; 1% SDS, Cat. #313-90275, Wako Pure Chemical Corporation; 10 mM EDTA pH 8.0, Cat. #311-90075, Wako Pure Chemical Corporation; Antifoam A, Cat. #A5633-25G, Sigma-Aldrich; 5 mM DTT, Cat. #048-29224, Wako Pure Chemical Corporation; 11.2 M 3-Mercapto-1,2-propanediol, Cat. #139-16452, Wako Pure Chemical Corporation; and DNase/RNase-free H2O, Cat. #10977015, Thermo Fisher Scientific, Waltham, MA, USA) (Ichihashi et al. 2020) was added to the sample, which was then homogenized by vortexing, followed by incubation at room temperature (22°C) for 5 min. The tube was centrifuged at 15,000 rpm for 10 min at room temperature (22°C), and the supernatant was transferred to a new 1.5 mL tube. Subsequently, 50 μL of LBB lysate was added to 1.5 mL tubes, and an equal amount of AMPure XP beads was added, followed by incubation at room temperature (22°C) for 5 min after vortexing. The mixture was placed on a magnetic station for 5 min and the supernatant was removed. The magnetic beads were washed twice with 200 μL of 80% ethanol. Finally, DNA was eluted with 20 μL of 10 mM Tris-HCl (pH 7.5).

16S rRNA gene amplicon sequencing

Library preparation using a two-step PCR amplification protocol has been reported in our previous publication (Kumaishi et al. 2021; Ichihashi et al. 2020). Briefly, the V4 region of the bacterial 16S rRNA gene was amplified using 515f and 806rB primers (forward primer: 5'- TCG TCG GCA GCG TCA GAT GTG TAT AAG AGA CAG- [3–6-mer Ns] – GTG YCA GCM GCC GCG GTA A -3'; reverse primer: 5'- GTC TCG TGG GCT CGG AGA TGT GTA TAA GAG ACA G [3–6-mer Ns] - GGA CTA CNV GGG TWT CTA AT -3') (Caporaso et al. 2011; Lundberg et al. 2013). Each sample (1 μL of 10-fold diluted DNA) was amplified in a 10 μL reaction volume containing 0.2 U KOD FX Neo DNA polymerase (TOYOBO Co., Ltd., Osaka, Japan), 2 × PCR buffer (TOYOBO Co., Ltd.), 0.4 mM dNTPs (TOYOBO Co., Ltd.), 0.2 μM forward and reverse primers, and 1 μM blocking primers (mPNA and pPNA; PNA BIO, Inc., Newbury Park, CA, USA). PCR was performed using the following conditions: 94°C for 2 min, followed by 35 cycles at 98°C for 10 s, 78°C for 10 s, 55°C for 30 s, 68°C for 50 s, and a final extension at 68°C for 5 min (ramp rate = 1°C/s).

The first PCR products were purified using a mixture of exonuclease and alkaline phosphatase. Two μL of ExoSAP-IT Express (Cat #75001.1.EA; Thermo Fisher Scientific) was added to 5 μL of the product obtained from the first PCR, and the mixture was incubated at 37°C for 4 min, followed by incubation at 80°C for 1 min.

The second PCR was conducted using the following primers: forward primer: 5'- AAT GAT ACG GCG ACC ACC GAG ATC TAC AC - [8-mer index] - TCG TCG GCA GCG TC -3', and reverse primer: 5'- CAA GCA GAA GAC GGC ATA CGA GAT - [8-mer index] - GTC TCG TGG GCT CGG -3' (Toju and Baba, 2018). Each sample (0.8 μL of purified product from the first PCR) was amplified in a 10 μL reaction volume containing 0.2 U KOD FX Neo DNA polymerase (TOYOBO Co., Ltd.), 2 × PCR buffer (TOYOBO Co., Ltd.), 0.4 mM dNTPs (TOYOBO Co., Ltd.), 0.3 μM forward and reverse primers, and 1 μM blocking primers (mPNA and pPNA). PCR was performed as follows: 94°C for 2 min, followed by eight cycles at 98°C for 10 s, 78°C for 10 s, 55°C for 30 s, 68°C for 50 s, and a final extension at 68°C for 5 min (ramp rate = 1°C/s). Following amplification, the PCR products for each sample were cleaned and size-selected using AMPure XP beads and washed twice with 80% ethanol. The libraries were eluted from the pellet with 10 μL of 10 mM Tris-HCl pH 7.5, quantified using a microplate photometer (Infinite 200 PRO M Nano+, TECAN Japan Co., Ltd., Kanagawa, Japan), and pooled into a single library in equal molar quantities. The pooled library was sequenced on an

Illumina MiSeq platform using a 2× 300-bp MiSeq Reagent Nano Kit v3 (Illumina, San Diego, CA, USA).

Taxonomic profiling and data preprocessing

Sequencing primers and short (<150 bp) reads were removed using Cutadapt v.1.18. The primer-free FASTQ files were further processed using R v3.6.0 with DADA2 v.1.14.0 (Callahan et al. 2016). First, the FASTQ file was trimmed, with a truncation length of 240 for forward reads and 160 for reverse reads. These reads underwent further quality filtering, as the error rates were calculated and removed from the duplicated reads. Forward and reverse sequences were merged. Next, potentially chimeric sequences and sequencing errors were removed. Taxonomy was assigned using the SILVA v.132 training set (Quast et al. 2012). The filtered matrix was rarefied to 3,000 reads per sample using the vegan 2.5-6 package (Oksanenvegan et al. 2020) and then used to calculate Bray-Curtis distance.

4 Reference

1. Bishop. C. M. 2006. Pattern Recognition and Machine Learning. Springer, 1 edition.

2. Blei, D and Jordan, M. 2006. Variational inference for Dirichlet process mixtures. Journal of Bayesian Analysis, 1:121–144.

3. Gopalan, P., Hao, W., Blei, D., Storey, J. 2016. Scaling probabilistic models of genetic variation to millions of humans. Nat Genet. 48, 1587-1590.

4. Hoffman, M., Blei, D., Wang, C. & Paisley, J. 2013. Stochastic variational inference. J. Mach. Learn. Res. 14, 1303–1347.

5. Jordan, M., Ghahramani, Z., Jaakkola, T. & Saul, L. 1999. Introduction to variational methods for graphical models. Mach. Learn. 37, 183–233.

6. Lartillot N. 2006. Conjugate Gibbs sampling for Bayesian phylogenetic models. J. Comput. Biol. 13:1701–1722.

7. Robbins, H. and Monro, S. 1951. A stochastic approximation method. The Annals of Mathematical Statistics 22, 400–407.

8. Wainwright, M. & Jordan, M. 2008. Graphical models, exponential families, and variational inference. Found. Trends Mach. Learn. 1, 1–305.

9. Kumaishi, K., Usui, E., Suzuki, K., Kobori, S., Sato, T., Toda, Y., Takanashi, H., Shinozaki, S., Noda, M.,Takakura, A., et al. 2021. Simple amplicon sequencing library preparation for plant root microbial community profiling. bioRxiv.

10. Ichihashi, Y., Date, Y., Shino, A., Shimizu, T., Shibata, A., Kumaishi, K., Funahashi, F., Wakayama, K., Yamazaki, K., Umezawa, A., et al. 2020. Multi-omics analysis on an agroecosystem reveals the significant role of organic nitrogen to increase agricultural crop yield. Proceedings of the National Academy of Sciences 117(25), 14552–14560.

11. Caporaso, J.G., Lauber, C.L., Walters, W.A., Berg-Lyons, D., Lozupone, C.A., Turnbaugh, P.J., Fierer, N., Knight, R. 2011. Global patterns of 16s rrna diversity at a depth of millions of sequences per sample. Proceedings of the national academy of sciences 108(Supplement 1), 4516–4522.

12. Lundberg, D.S., Yourstone, S., Mieczkowski, P., Jones, C.D., Dangl, J.L. 2013. Practical innovations for high-throughput amplicon sequencing. Nature methods 10(10), 999–1002.

13. Callahan, B.J., McMurdie, P.J., Rosen, M.J., Han, A.W., Johnson, A.J.A., Holmes, S.P. 2016. Dada2: high-resolution sample inference from illumina amplicon data. Nature methods 13(7), 581–583.

14. Quast, C., Pruesse, E., Yilmaz, P., Gerken, J., Schweer, T., Yarza, P., Peplies, J., Glockner, F.O. 2012. The silva ribosomal rna gene database project: improved data processing and web-based tools. Nucleic acids research 41(D1), 590–596.

15. Oksanen, J., Blanchet, F., Friendly, M., Kindt, R., Legendre, P., McGlinn, D., et al. 2020: vegan: community ecology package. R package version 2.5-7.
