# Supplementary figures and images for: Stochastic variational variable selection for high-dimensional microbiome data
Source: Microbiome. 2022 Dec 24;10:236. doi: 10.1186/s40168-022-01439-0 (PMC9789572; doi:10.1186/s40168-022-01439-0)

## Supplementary Figures

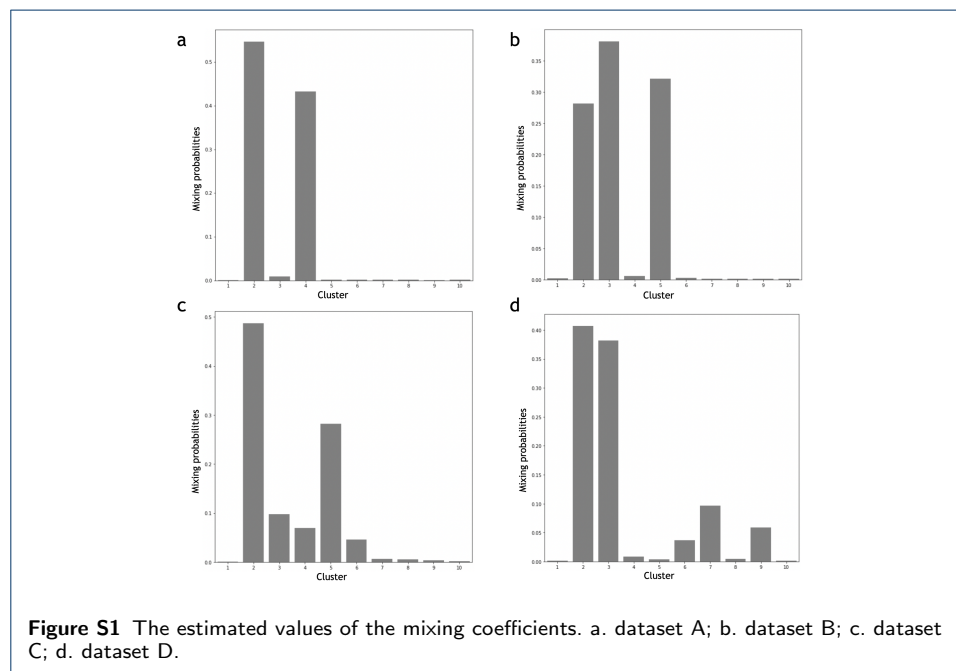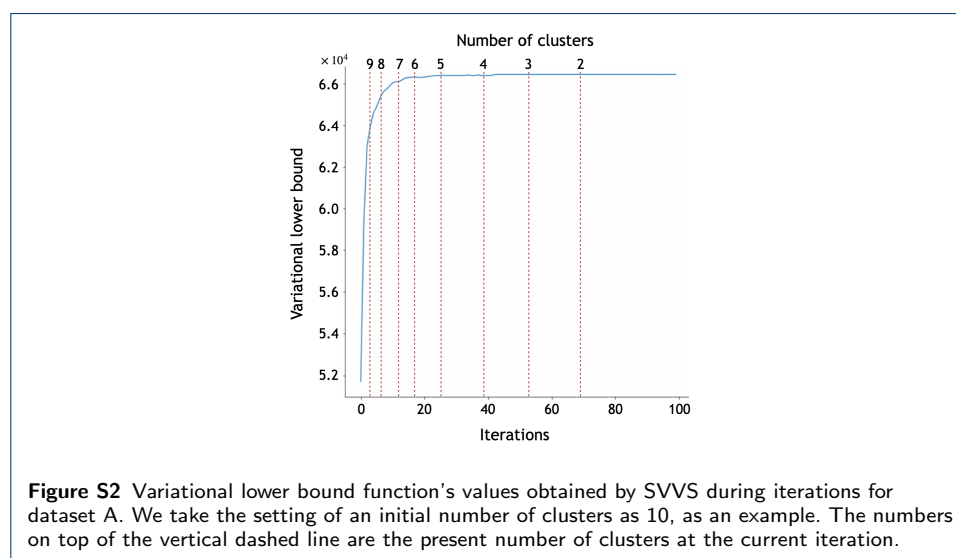

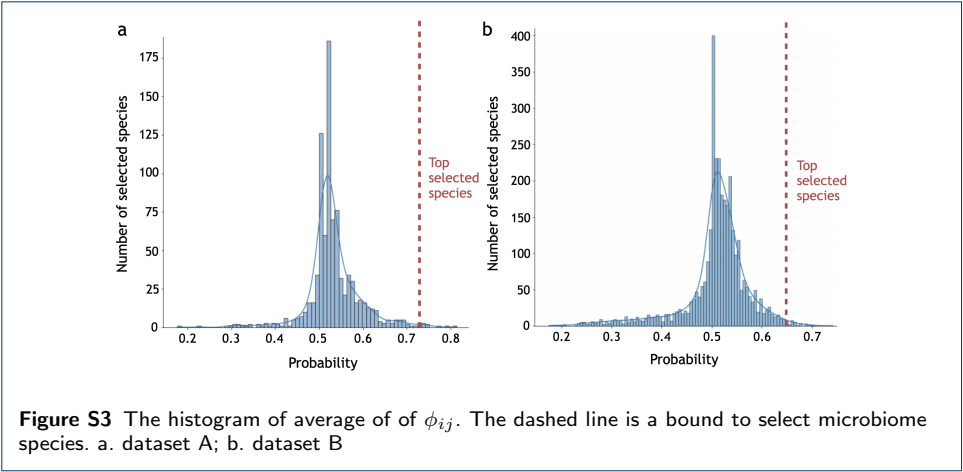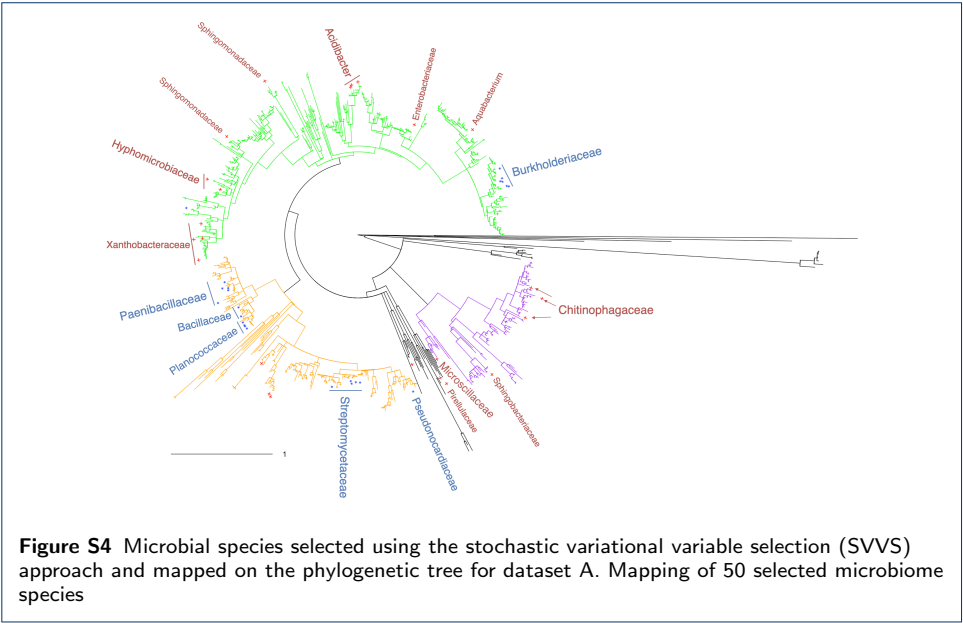

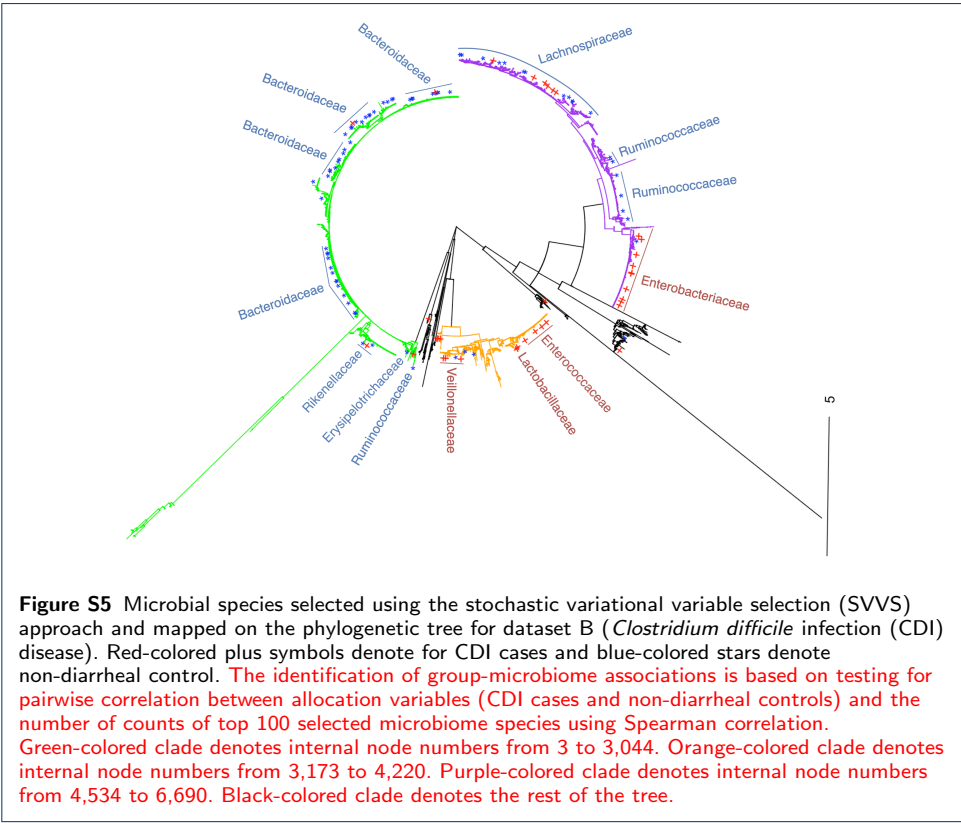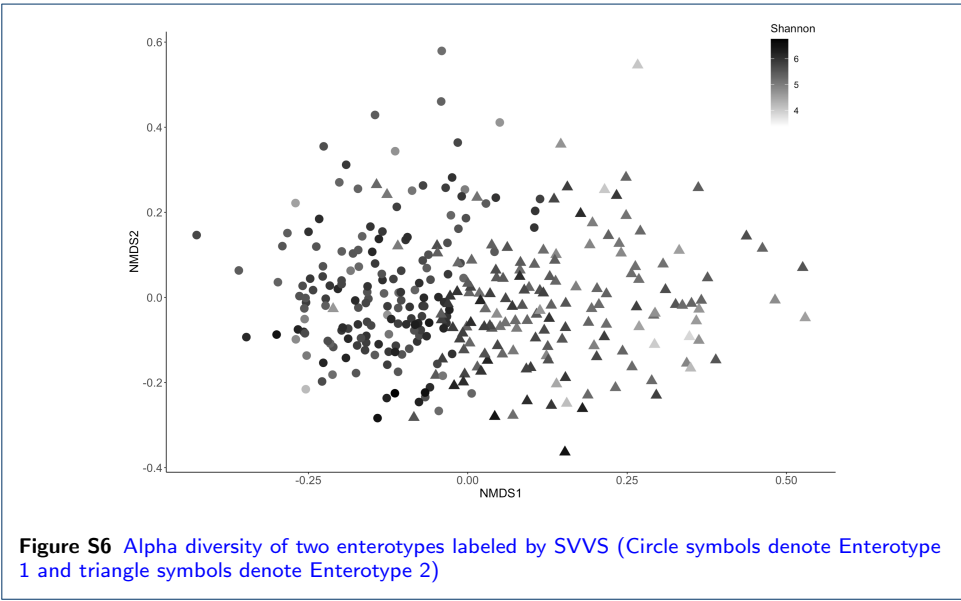

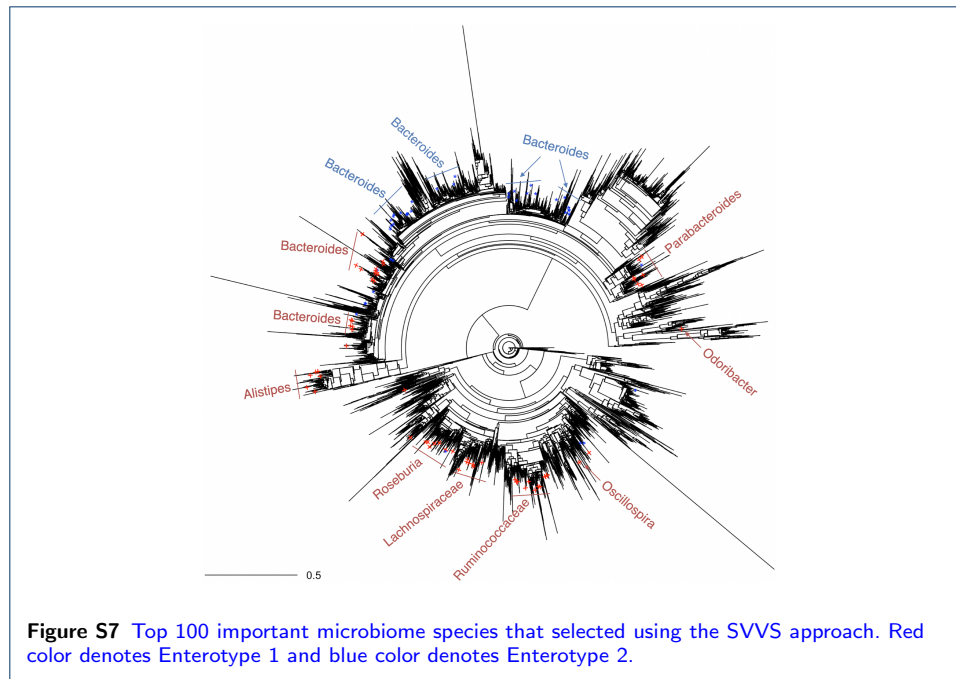

Supplement: Supplementary file 3 — Additional file 2: Figure S1. The estimated values of the mixing coefficients. a. dataset A; b. dataset B; c. dataset C; d. dataset D. Figure S2. Variational lower bound function values obtained by SVVS during iterations for dataset A. We take the setting of an initial number of the clusters as 10, as an example. The numbers on top of the vertical dashed line are the present number of clusters at the current iteration. Figure S3. Histogram of the average of $\phi_{ij}$ϕij. The dashed line is a bound to select microbiome species. a. dataset A; b. dataset B. Figure S4. Microbial species selected using the stochastic variational variable selection (SVVS) approach and mapped on the phylogenetic tree for dataset B (Clostridium difficile infection (CDI) disease). Red-colored plus symbols denote for CDI cases and blue-colored stars denote non-diarrheal control. Figure S5. a. Alpha diversity of two enterotypes labeled by SVVS (Circle symbols denote Enterotype 1 and triangle symbols denote Enterotype 2); b. Top 100 important microbiome species that selected using the SVVS approach. Red color denotes Enterotype 1 and blue color denotes Enterotype 2. [file 40168_2022_1439_MOESM2_ESM.pdf]
